# Screen Culture’s Association with Generation Z Adolescents’ Posture and Well-Being in Egypt: Cross-Sectional Study

**DOI:** 10.3390/healthcare14203498

**Published:** 2026-10-18

**Authors:** Alaa Noureldeen Kora, Yousef M. Alshehre

**Affiliations:** Department of Health Rehabilitation Sciences, Faculty of Applied Medical Sciences, University of Tabuk, Tabuk 71491, Saudi Arabia; yalshehre@ut.edu.sa

**Keywords:** screen time, Generation Z, adolescents, insomnia, postural habits, quality of life

## Abstract

**Highlights:**

**What are the main findings?**

- Most Egyptian Gen Z adolescents spend more than the recommended duration on screens, with the highest recreational use during weekends and the lowest during workdays.
- Activity-specific screen-use models adjusted for demographic and anthropometric characteristics explained limited variability in insomnia, postural habits, and health-related quality of life.

**What are the implications of the main findings?**

- Interventions should target the specific types and functions of screens, rather than the total amount of screen time, since weekday academic screen use is more closely related to insomnia than weekend leisure screen use.
- Future research should use multi-factorial models including psychological, social and lifestyle factors in addition to screen time to better understand adolescent well-being.

**Abstract:**

**Background:** Egyptian Generation Z adolescents are heavily engaged with screens for several purposes, including education, entertainment, social media, and gaming. This raises red flags about how screen culture may affect sleep quality, posture-related habits, and health-related quality of life. **Objectives:** To examine how different types and amounts of weekday and weekend screen time are associated with symptoms of insomnia, body awareness of postural habits, and health-related quality of life among Egyptian Generation Z adolescents. **Materials and Methods**: A cross-sectional English-language online survey was completed by a self-selected convenience sample of Egyptian adolescents aged 13–18 years from multiple governorates; 530 participants were included in the analysis. Participants reported total screen time, the Insomnia Severity Index, Body Awareness of Postural Habits questionnaire, and the Pediatric Quality of Life Inventory. Multiple linear regression models were adjusted for age, gender, body mass index, level of education, and place of residence. **Results:** After false-discovery-rate adjustment across 30 comparisons, 13 activity-specific associations met the significance threshold. Higher ISI scores were associated with weekday job or internship-related use, weekday and weekend entertainment use, and weekday and weekend social-media use. Lower Q-BAPHYP scores were associated with weekday and weekend entertainment and social-media use. Lower PedsQL scores were associated with weekday job or internship-related use, weekday entertainment use, and weekday and weekend social-media use. The models explained limited variability in the outcomes. **Conclusions:** The screen use of Egyptian Generation Z adolescents is associated with insomnia, postural awareness, and overall quality of life; additional unmeasured factors probably influence such outcomes as well.

## 1. Introduction

Screen culture refers to the ways digital media are integrated into adolescents’ daily lives, including the purposes and contexts of use [1]. It differs from screen time, which describes the duration of digital device use [2]. Considering screen time alone may therefore overlook differences between activities that form part of adolescents’ routines [1,2].

Digital media are used for learning, communication, and recreation. Screen use increased during the COVID-19 pandemic, when school closures and distancing measures shifted many activities online [3]. These changes reinforced the role of screens in adolescents’ everyday lives, while also making it useful to examine how screens are used alongside how long they are used [1,2,3].

Studies of adolescent screen use have documented substantial daily exposure [4]. Yet a single measure of total screen time cannot show whether time is spent on school-related or recreational activities, or whether use differs between weekdays and weekends [1,2]. Examining these patterns separately may provide a clearer basis for studying their associations with adolescent health and well-being.

Generation Z, commonly known as Gen Z, is a generation of young individuals born between 1997 and 2012. They are known as the original “digital natives”. They are the first generation who have never known a world without the internet. They swipe, tap, and scroll on their smartphones and tablets with the same unconscious ease as breathing. Gen Z are not just users of technology; they are masters at using screens for learning, socializing, and entertainment [5,6]. Some researchers take it a step further and call Gen Z “the mobile natives.” Gen Z is different from older generations in more ways than just how comfortable they are with technology. Since they were very young, they have always had pocket-sized screens that were connected to the internet. This constant closeness may be associated with how they see themselves, make friends, and spend their free time. On the other hand, some studies indicate that they may find it difficult to distinguish between online and offline life, which may make it hard for them to make real friends and manage their time [6]. This generational shift is particularly evident in Egypt, as since the mid-2000s, the internet and smartphones have spread rapidly in Egypt, making digital technology part of adolescents’ daily lives. More than 100 million people in Egypt use mobile phones, and a lot of them are adolescents. They spend several hours per day online, usually through their smartphones [7,8]. Daily internet usage is not just about entertainment or social connection; it has also shown a broader shift toward digital education, especially after school closures during the COVID-19 pandemic, when students had to turn to online platforms and mobile apps for e-learning, making screens an essential part of their academic life [7,8]. Adolescents in Egypt enjoy social media and video games, and it has been reported that they engage heavily with social media platforms such as WhatsApp, Instagram, TikTok, Facebook, and X, as well as online gaming. Previous research in Egyptian adolescent and university samples has reported problematic patterns of internet and smartphone use [8,9]. These findings concern problematic use rather than screen-use duration alone. So, the key finding is clear: entertainment, social connections, and learning among Egyptian adolescents are now revolving around screens, making digital devices just part of their typical day [8,9].

Excessive screen use among adolescents has been consistently associated with a range of health concerns, most notably sleep disturbances, musculoskeletal complaints, and diminished health-related quality of life [10,11,12]. Screen usage is frequently linked with inadequate sleep in adolescents, characterized by reduced sleep length, delayed bedtimes, prolonged time to go to sleep, reduced sleep quality, and increased daily fatigue, particularly when screens are utilized at bedtime or during nocturnal hours [10]. A 2024 panel by the National Sleep Foundation concluded that screen usage is associated with poorer sleep health in adolescents, who engage at the pre-sleep stage in stimulating and interactive screen content, including social media, gaming, and video streaming, which may disrupt sleep timing, duration, and quality [13].

Most adolescents spend several hours daily in prolonged seated or slouching positions, in addition to intensive use of handheld smart devices for school, work, or leisure. This raises concerns related to posture and musculoskeletal strain in adolescents, as recent studies link extended sitting and poor device-use posture, such as forward head, rounded shoulders, and reduced lumbar curve, to neck, upper- and lower-back discomfort, and abnormal spinal alignment [14,15]. Researchers have also found associations between excessive screen and handheld device usage and physical complaints, particularly neck and lower back pain. A primary concern is that adolescents often maintain a forward-bent neck and slouched posture while using technology, which may exert continuous stress on their developing muscles and spines [11,12].

Health-related quality of life (HRQoL) in adolescents is a multidimensional construct encompassing their subjective perceptions of physical health, emotional and psychological well-being, social relationships, and functioning in key environments such as family, peer groups, and school [16,17,18]. These domains demonstrate how adolescents perceive their bodies (e.g., pain, energy, physical functioning), manage emotions and stress, engage socially and perceive support, and fulfill academic expectations. Consequently, HRQoL serves as a central indicator of overall well-being and future health outcomes [16,18,19].

There is growing evidence that sedentary behaviors associated with screens, particularly time spent on video games, cell phones, and tablets, are associated with diminished HRQoL. Extended screen time is associated with reduced overall HRQoL scores and specific declines in psychological well-being, peer social support, and the school environment. Furthermore, prolonged daily screen exposure is associated with lower physical and psychosocial HRQoL among substantial adolescent cohorts [17,19,20].

Some research studies relevant for Middle East and North Africa (MENA) adolescents regarded screen exposure merely as total daily screen time. While useful, this approach provides limited insight into whether different activities—such as social media, gaming, video streaming, or digital schoolwork—are differentially associated with outcomes. Nor does it capture how screen use might vary between weekdays and weekends. Thankfully, recent studies have begun to disaggregate time spent on television, electronic gaming, computers, and social media. Their results showed that distinct screen types showed different dose–response relationships with outcomes such as sleep quality, musculoskeletal pain, life satisfaction, and mental health. Even so, most of this research focuses on recreational screen use, neglecting other forms of screen engagement and the distinction between weekday and weekend use [21,22].

As a result, the current study examines subtype-specific screen use and contrasts patterns between weekdays and weekends to identify which patterns are most strongly associated with adverse outcomes for adolescents at risk. The majority of existing research evidence assessing screen behaviors and health outcomes comes from high-income regions, specifically Europe and North America. Very few studies have focused on adolescents in the MENA region, even though the region is rapidly converting to digitalization, and many young individuals already exceed 2 h of daily screen time [23]. This is why generating detailed, locally relevant evidence from Egyptian adolescents is crucial. If such evidence is not available, public health and school-based programs may have to rely on results from very different cultural and digital settings. Recommendations can be created that are not only practicable and relevant for Egyptian adolescents but also evidence-based by comprehending screen type, timing, and use within Egypt’s own sociocultural context and ongoing digital transition.

Screen culture encompasses the purposes, contexts, and patterns of digital engagement. This study examines one measurable aspect of that broader concept: self-reported activity-specific screen-use duration on weekdays and weekends. Study- or school-related activities, job- or internship-related activities, entertainment, gaming, and social media were examined because they represent different purposes of screen use. Weekdays and weekends were considered separately because educational demands and daily routines may differ. The study did not measure screen culture as a validated composite construct or assess problematic or addictive screen use.

Consequently, this study aimed to assess associations between weekday and weekend screen-time activities and insomnia symptoms, body awareness of postural habits, and health-related quality of life among Egyptian adolescents aged 13–18 years. The analyses evaluated the null hypothesis of no association between each weekday or weekend activity-specific screen-use duration and each outcome after adjustment for the specified covariates.

## 2. Materials and Methods

### 2.1. Study Setting, Participants, and Data Collection

Adolescents aged 13–18 years attending schools or universities across multiple Egyptian governorates were recruited using self-selected convenience sampling. Potential participants were approached individually and invited to complete an English-language online questionnaire. Eligible adolescents who agreed to participate received the survey link. Schools and classes were not selected as sampling units, and no probability-based sampling procedure was used. Recruitment included rural, urban, and mixed residential settings, providing geographic diversity without establishing national representativeness. The questionnaire link was distributed to approximately 2000 potential participants. A total of 533 questionnaires were submitted, yielding an estimated response rate of approximately 27%. Following exclusions, 530 participants were included in the analyses. The estimated response rate was calculated using submitted questionnaires and the approximate number of potential participants reached; the exact recruitment denominator was not recorded.

Before completing the questionnaire, participants confirmed that they could read and understand the questions in English. No formal English proficiency test was administered. They were also required to use at least one screen device, such as a smartphone, laptop, or tablet, often.

The online survey first presented the study information and consent form to the parent or legal guardian. Parental consent was recorded electronically; however, the identity of the person providing consent was not independently authenticated. Only after the parent or guardian provided consent was the questionnaire made available to the adolescent. The adolescent then reviewed the assent form and indicated whether they agreed to participate before answering the study questions. Participation was voluntary, and adolescents could stop completing the questionnaire at any time before submission without providing a reason.

All participants completed an electronic assent form online, and for adolescents, parental or legal guardian consent was also acquired online before taking the survey. Data were collected from the beginning of July 2025 until the end of January 2026. An online questionnaire was designed with Google Forms to make it easy for participants to access.

### 2.2. Sample Size

Sample size was calculated a priori using G*Power 3.1 for multiple linear regression with 10 predictors, an effect size of f^2^ = 0.08, α = 0.05, and power = 0.95, yielding a minimum requirement of 335 participants. The recruitment target was increased by 15% to approximately 385 participants to allow for incomplete or invalid responses. Following exclusion of two records without consent and one duplicate, 530 participants were included in the revised analyses. Each revised regression model included one continuous screen-use measure and adjustment for age, sex, residence, educational level, and continuous BMI. The original calculation served as a recruitment-planning estimate; it did not account for multiplicity across the 30 activity–outcome comparisons and did not establish adequate power for every individual association or sensitivity analysis. The analyses are therefore interpreted as exploratory, with emphasis on effect estimates, confidence intervals, and false-discovery-rate-adjusted *p*-values.

### 2.3. Instrumentation

All instruments were administered in English. The ability to read and understand English was an eligibility criterion, assessed as described in Section 2.1.

#### 2.3.1. Screen Time Questionnaire

The Questionnaire for Screen Time of Adolescents (QueST) is a self-administered instrument developed to assess habitual screen time across four activity-based constructs: (1) job-related activities (including both studying/homework and work/internship tasks); (2) watching videos (e.g., TV shows, movies, news, sports); (3) playing video games; and (4) using social media and chat applications. Each construct is reported separately for weekdays and weekend days. QueST showed high Content validity (S-CVI = 0.94 and 0.98) and a fair to excellent Test–retest reliability when tested one week later in adolescents (ICCs ranging from 0.24 to 0.76), varying by activity and day type [24].

Study or school-related screen time and job or internship-related screen time were recorded separately. The questionnaire did not distinguish use during teacher-led lessons from use outside lessons or at home. The existing study/school-related classifications—typical, balanced, and excessive—are reported descriptively in Supplementary Table S1. Their application was inconsistent at exactly 2 h/day on weekdays; therefore, these labels were not treated as consistently applied thresholds and were excluded from the revised regression analyses. All ten activity-specific screen-use durations were modeled continuously. The questionnaire did not record whether these activities occurred during lessons, outside school hours, or at home. For the present analyses, study or school-related use, job or internship-related use, entertainment, gaming, and social-media use were recorded separately for weekdays and weekends.

#### 2.3.2. Insomnia Severity Index

The Insomnia Severity Index (ISI) is a brief self-report questionnaire that evaluates the type, severity, and adverse effects of insomnia experienced in the preceding month. There are seven questions on the ISI that look at problems with falling asleep, staying asleep, and waking up early; sleep satisfaction; how sleep problems affect daytime functioning; how visible sleep problems are; and how distressing they are. Each item is rated on a scale of 0 to 4, with a total score ranging from 0 to 28, with higher scores indicating more severe insomnia [25,26].

The ISI showed excellent to exceptional internal consistency (Cronbach’s α generally ~0.79–0.92; pooled α = 0.83 in a 33-study meta-analysis) and strong test–retest reliability (ICC ≈ 0.74–0.90) [25,27,28], making it used extensively in both clinical and research settings [26].

#### 2.3.3. Body Awareness of Postural Habits Questionnaire

The Questionnaire on Body Awareness of Postural Habits in Young People (Q-BAPHYP) is a self-report questionnaire that was designed to assess adolescents’ awareness of their postural habits in everyday contexts [29]. The Q-BAPHYP is divided into four dimensions: postural habits in the classroom, at home, when carrying objects, and teachers’ disciplinary orientations, with a total of 35 items. Each item is answered, then rated with one of the following responses: 1 = Never; 2 = Rarely; 3 = Frequently; 4 = Always; 5 = Don’t know/don’t remember. Following the instrument’s scoring protocol, responses are recoded to values from −2 to +2, where negative values indicate more adequate (healthier) postural habits and positive values indicate less adequate habits; the “Don’t know/don’t remember” option is treated as a neutral response (0). A total score is obtained by summing all recoded item scores. Consequently, higher total scores reflect poorer postural awareness, while lower (including negative) scores reflect better postural awareness [30].

The Q-BAPHYP shows strong content validity (72% expert agreement) and good internal consistency (Cronbach’s α = 0.80). Test–retest reliability is acceptable for most dimensions (ICCs = 0.59–0.74), supporting its use for assessments in adolescents [29,30].

#### 2.3.4. Pediatric Quality of Life Inventory (PedsQL) Teen Report

The PedsQL 4.0 Teen self-report (13–18 years) is a brief, well-validated tool that consists of 23 items, suitable for both clinical and research applications across adolescent populations. It is a multidimensional measure of HRQoL that assesses physical, emotional, social, and school functioning domains [31].

The PedsQL psychometric properties in adolescent populations are strong, with high internal consistency (Cronbach’s α ≥ 0.80), good test–retest reliability (intraclass correlations in the acceptable to excellent range), and construct and convergent validity, as supported by moderate to strong correlations with functional impairment and other HRQoL measures [31].

### 2.4. Data Analysis and Statistical Design

All statistical analyses were performed using SAS version 9.4 through SAS^®^ OnDemand for Academics (SAS Institute Inc., Cary, NC, USA), Python 3.13.14 and statsmodels version 0.14.6. The available dataset contained 533 records. One record without consent and two duplicates were excluded, leaving 530 participants. All retained participants had complete data for the demographic covariates, ten screen-use measures, and three outcome totals; therefore, no additional participants were excluded because of missing model variables, and no imputation was performed.

Descriptive statistics (means, standard deviations, medians, IQR, and percentages) were undertaken to characterize participants’ demographic characteristics, patterns of screen use, insomnia severity, bodily awareness of postural behaviors, and health-related quality of life (PedsQL).

Multiple linear regression was used for primary analyses to assess the associations between screen time factors (predictors) and the three primary outcome measures: total ISI score, total Body Awareness of Postural Habits score (Q-BAPHYP), and total PedsQL score.

Thirty separate multiple linear regression models examined the associations between ten screen-use measures—study- or school-related activities, job- or internship-related activities, entertainment, gaming, and social media, each recorded for weekdays and weekends—and three outcome totals: ISI, Q-BAPHYP, and PedsQL. Each model included one continuous screen-use measure and adjustment for age, sex, continuous BMI, educational level, and residence. All specified adjustment covariates were retained regardless of statistical significance; no significance-based variable selection or stepwise model reduction was performed. Continuous BMI was retained without BMI severity categories to avoid including two representations of the same construct. Screen-use classifications were not included in the regression models.

ISI, Q-BAPHYP, and PedsQL total scores were the three main outcomes. The revised activity-specific analytical framework was exploratory and was developed during manuscript revision, rather than prespecified before data collection. All ten activity-period measures were evaluated consistently against each outcome, yielding 30 exposure–outcome comparisons. Because reported activity durations may overlap, each model included one screen-use measure rather than entering all activities simultaneously. These models estimate associations adjusted for demographic and anthropometric covariates, but not independent of other screen-use activities. All 30 estimates are reported, with FDR adjustment across the comparisons. Alternative outcome models, functional-form comparisons, and influence and extreme-value analyses were supplementary exploratory assessments.

Multiple linear regression was retained to estimate adjusted mean differences in outcome scores per additional hour/day of screen use. Normality of the outcome variables or predictors was not treated as a prerequisite for linear regression. HC3 heteroscedasticity-robust standard errors were used for inference; however, these do not correct an incorrectly specified functional form. Linear and spline specifications were compared for each activity–outcome pair, and sensitivity analyses examined the influence of observations flagged by Cook’s distance. Alternative outcome models were also fitted to assess the sensitivity of findings to the outcome-model specification.

Residual-versus-fitted plots and normal Q–Q plots were examined for all 30 adjusted linear regression models. Residual-versus-fitted plots included a locally weighted smoother to assess systematic patterns and changes in residual spread. Q–Q plots assessed departures from a normal residual distribution, particularly in the tails. These assessments were considered alongside exposure-specific linear-versus-spline comparisons, influential-observation analyses, and alternative outcome models. Normality of the outcome variables themselves was not used to determine the suitability of linear regression.

Screen-use activities were examined separately because their reported durations may overlap. This specification estimates the association of each activity with an outcome after adjustment for the demographic and anthropometric covariates; it does not estimate an association independent of other screen-use activities. Coefficients represent the adjusted outcome-score difference per additional hour/day. HC3 robust standard errors were used to calculate 95% confidence intervals, and Benjamini–Hochberg false-discovery-rate adjustment was applied across the 30 screen-use associations.

The Q-BAPHYP total score was retained as the primary postural outcome to examine overall reported postural habits. The total score was selected to address the study question concerning overall postural habits. Separate dimension-specific regression models were not undertaken, limiting additional outcome comparisons. Component-level descriptive statistics are presented separately in Supplementary Table S3.

Statistical significance for the main screen-use associations was evaluated using Benjamini–Hochberg false-discovery-rate-adjusted *p*-values below 0.05 across the 30 activity–outcome comparisons. Unadjusted *p*-values and pointwise 95% confidence intervals are also reported. *p* values between 0.05 and 0.10 were not treated as a separate category of significance. Alternative outcome analyses, functional-form comparisons, and sensitivity refits were exploratory; their *p*-values were not adjusted for multiplicity and were not used to identify additional statistically significant main associations.

This study is described according to the Strengthening the Reporting of Observational Studies in Epidemiology (STROBE) criteria for cross-sectional studies.

## 3. Results

### 3.1. Demographic Characteristics of the Sample

The analysis included 530 Egyptian adolescents aged 13–18 years. All retained participants had complete demographic, screen-use, and outcome data for the adjusted analyses. The mean age was 15.98 years (SD = 1.50), and females and males each accounted for 50.0% of the sample. Mean BMI was 23.18 kg/m^2^ (SD = 3.94). Participants were recruited from 25 governorates; 48.7% lived in rural settings, 26.2% in urban settings, and 25.1% in mixed settings. Most participants attended secondary school (61.1%; Table 1).

### 3.2. Descriptive Analysis of Screen Time

Median study or school-related screen time was 4.0 h/day on both weekdays and weekends. Median job or internship-related use was 3.0 h/day on weekdays and 2.0 h/day on weekends. Entertainment, gaming, and social-media use had higher weekend medians: 4.0 versus 3.0 h/day, 3.0 versus 2.0 h/day, and 4.0 versus 3.0 h/day, respectively (Table 2). These comparisons describe the weekday and weekend distributions without testing within-participant differences. The study/school-related screen use classifications are presented in Supplementary Table S1.

Seventeen participants (3.2%) reported 24 h/day for at least one activity. Activity-specific reports were summarized separately and were not summed. Descriptive summaries by age and educational level are provided in Supplementary Table S2.

### 3.3. Descriptive Analysis of Outcome Measures

The median ISI score was 12 (Q1–Q3, 9–15; range, 0–23). No clinically significant insomnia was reported by 91 participants (17.2%), subthreshold insomnia by 297 (56.0%), moderate insomnia by 130 (24.5%), and severe insomnia by 12 (2.3%). Overall, 142 participants (26.8%) had moderate or severe insomnia (ISI ≥ 15).

The median Q-BAPHYP total score was 1 (Q1–Q3, −6 to 8; range, −42 to 46). Higher scores indicate poorer postural habits, whereas lower scores indicate more favorable postural habits. Component-level summaries are provided in Supplementary Table S3.

The mean PedsQL total score was 65.93 (SD = 13.68). Social functioning had the highest mean score (71.89), followed by physical functioning (67.15), school functioning (64.51), and emotional functioning (59.45; Table 3).

### 3.4. Multiple Linear Regression Analysis

Thirty separate multiple linear regression models examined the ten weekday and weekend screen-use measures in relation to the three outcome totals. Each model included one screen-use measure and adjusted for age, sex, residence, educational level, and continuous BMI. HC3 robust standard errors were used, and Benjamini–Hochberg false-discovery-rate (FDR) adjustment was applied across the 30 screen-use associations. Each model included 530 participants.

Thirteen associations met the FDR-adjusted significance threshold of 0.05. Table 4, Table 5 and Table 6 report all screen-use coefficients, including associations that did not meet this threshold.

#### 3.4.1. Insomnia Model

Higher ISI scores were associated with weekday job or internship-related use, weekday and weekend entertainment use, and weekday and weekend social-media use after FDR adjustment. Adjusted differences ranged from 0.161 to 0.232 ISI points per additional hour/day. Study or school-related use, gaming use, and weekend job or internship-related use did not meet the FDR-adjusted significance threshold.

The adjusted R^2^ values ranged from 0.059 to 0.092, indicating that the models explained a limited proportion of the variability in insomnia scores (Table 4).

#### 3.4.2. Body Awareness of Postural Habits in Young People Model

Weekday and weekend entertainment and social-media use were associated with lower Q-BAPHYP total scores after FDR adjustment. Adjusted differences ranged from −0.414 to −0.254 points per additional hour/day, indicating more favorable reported postural habits. The remaining associations did not meet the FDR-adjusted significance threshold.

The adjusted R^2^ values ranged from 0.006 to 0.021, indicating very limited explained variability in postural-habit scores (Table 5).

#### 3.4.3. Pediatric Quality of Life (PedsQL) Model

Lower PedsQL total scores were associated with weekday job or internship-related use, weekday entertainment use, and weekday and weekend social-media use after FDR adjustment. Adjusted differences ranged from −0.574 to −0.471 points per additional hour/day. The remaining associations did not meet the FDR-adjusted significance threshold.

The adjusted R^2^ values ranged from 0.037 to 0.060, indicating that the models explained a limited proportion of the variability in health-related quality of life (Table 6).

### 3.5. Regression Diagnostics and Sensitivity Analyses

Residual-versus-fitted and normal Q–Q plots were examined for all 30 adjusted models (Supplementary Figures S1–S6). ISI and PedsQL residuals broadly followed the normal reference in the central portions of the Q–Q plots, with departures in the tails and modest local changes in residual spread. Q-BAPHYP models showed more pronounced tail departures, compatible with heavier-than-normal residual tails, and occasional large residuals. Residual-versus-fitted plots did not show a pronounced common funnel pattern across the central fitted ranges, although local curvature and variation in spread remained. These findings were considered alongside the functional-form and sensitivity analyses and were not interpreted as establishing that all linear regression assumptions were satisfied.

Alternative outcome models were fitted for all 30 activity–outcome pairs using binary logistic regression for moderate or severe insomnia (ISI ≥ 15) and median quantile regression for Q-BAPHYP and PedsQL. Nine of ten estimates for each outcome had the same direction as the corresponding linear-regression coefficient, although many confidence intervals included the null. The linear coefficients for associations showing evidence compatible with nonlinearity should be interpreted as overall linear summaries rather than constant differences across the full exposure range. Among the associations meeting the main FDR-adjusted significance threshold, alternative-model intervals excluded the null for weekday job or internship-related use, weekend entertainment, and weekend social-media use with insomnia; weekday social-media use with Q-BAPHYP; and weekday entertainment and weekday and weekend social-media use with PedsQL (Supplementary Table S4). These models assess different features of the outcome distributions.

Linear-versus-spline comparisons suggested nonlinearity for weekday and weekend entertainment use with Q-BAPHYP (unadjusted *p* = 0.036 and *p* <0.001, respectively) and weekend job or internship-related use with PedsQL (*p* = 0.006). These exploratory comparisons were not adjusted for multiplicity (Supplementary Table S5). The residual assessments did not remove these exposure-specific functional-form concerns; the corresponding linear coefficients should therefore be interpreted as overall linear summaries rather than constant differences across the full exposure range.

Excluding observations flagged by Cook’s distance, the direction of all 13 associations meeting the main FDR-adjusted significance threshold was preserved. The confidence interval for weekend social-media use with Q-BAPHYP included zero after exclusion, whereas the other 12 corresponding intervals excluded zero (Supplementary Table S6).

Replacing 24 h reports with 20 h/day in the affected models preserved coefficient directions and changed coefficients by no more than 0.028 outcome points per hour. The pointwise sensitivity intervals for all 13 associations meeting the main FDR-adjusted threshold continued to exclude zero (Supplementary Table S7). These sensitivity comparisons were not subjected to a new FDR adjustment.

## 4. Discussion

This study identified activity-specific associations between weekday and weekend screen use and insomnia symptoms, self-reported postural habits, and health-related quality of life among participants in this self-selected sample of Egyptian adolescents. After FDR adjustment, weekday job or internship-related use, weekday and weekend entertainment use, and weekday and weekend social-media use were associated with higher insomnia scores. Weekday and weekend entertainment and social-media use were associated with lower Q-BAPHYP scores; however, this finding does not establish a beneficial relationship between these activities and postural habits. Lower health-related quality-of-life scores were associated with weekday job or internship-related use, weekday entertainment use, and weekday and weekend social-media use. Study or school-related use and gaming did not meet the FDR-adjusted significance threshold for any outcome. These findings represent small estimated score differences; meeting the FDR-adjusted significance threshold does not establish clinical importance. These findings describe activity-specific patterns of association across the three adolescent health outcomes. These associations were adjusted for demographic and anthropometric characteristics, but not for other screen-use activities, and should not be interpreted as independent activity-specific relationships.

### 4.1. Screen Time Patterns

Participants reported screen use for educational, occupational, and recreational activities on both weekdays and weekends. Median reported durations for entertainment, gaming, and social-media use were higher on weekends than on weekdays. These estimates describe individual activities rather than total daily screen time, because activity durations may overlap and were not summed.

Previous studies also describe substantial screen use among adolescents, although differences in measurement limit direct comparisons with the activity-specific estimates reported here. A global systematic review of children and adolescents aged 6 to 14 found that they spent an average of 2.77 h a day on screens, with 46 to 59% spending at least 2 h a day on screens. The main activities were entertainment and using social media, as well as e-learning. These activities were performed using TVs, computers, and mobile devices. Worldwide, researchers identified excessive screen time as a “common behavior” [32].

A comprehensive scoping review of 130 surveillance studies revealed that, on average, 52% of participants exceeded 2 h of screen time per day, with total screen usage averaging approximately 3.6 h daily and numerous adolescents reaching 4–5+ hours; screens were characterized as prominent in the daily lives of adolescents [33]. However, screen-time estimates from other countries vary, and some adolescent samples report higher use than observed here; direct comparisons require caution because studies differ in age range, sampling, and how screen time is measured.

More complex patterns involving weekdays, weekends, and activities reinforce the idea that weekends are primarily for entertainment. The Adolescent Brain Cognitive Development (ABCD) Study demonstrated that self-reported screen time was significantly higher on weekends than on weekdays, with variations in usage patterns across television/movies, gaming, and online social interactions [34]. Furthermore, an additional ABCD analysis revealed that adolescents engage more in screen time, video gaming, and online social interactions on weekends, with device-specific behaviors (gaming versus social networking) varying by gender, highlighting the dual functions of screens in recreation and communication [4]. Altogether, these studies line up with current findings on adolescents’ increased screen exposure on weekdays and weekends, emphasizing the presence of recreational use—especially on weekends—alongside academic or job-related use that sustains throughout the week.

### 4.2. Insomnia

The adjusted insomnia models explained 5.9–9.2% of the variability in ISI scores. Although several screen-use associations met the FDR-adjusted significance threshold, their estimated per-hour differences were small, and statistical significance alone does not establish clinical importance. After FDR adjustment, higher ISI scores were associated with weekday job or internship-related use, weekday and weekend entertainment use, and weekday and weekend social-media use. For weekday job or internship-related use, the estimated difference was 0.218 ISI points per additional hour/day (95% CI: 0.109–0.327). This was a small estimated score difference, and its clinical importance remains uncertain. The observed associations do not establish whether screen use preceded insomnia symptoms or whether adolescents with insomnia engaged in different screen-use patterns.

Previous research also suggests that associations between screen use and insomnia vary by activity and timing. A Chinese longitudinal study involving 831 adolescents, assessed by the ISI, showed that the impact of screen time varied by duration and activity: daytime television viewing was linked to increased insomnia severity, while nighttime work- or study-related screen time was associated with decreased insomnia severity [35].

Additional studies on university students also suggested that overall weekend screen use had little effect on insomnia symptoms. Weekend productivity and video game screen time predicted higher ISI scores, while weekday screen time was not significantly associated, highlighting the relevance of non-work behaviors and weekends, but the variation explained was minimal [36]. This contradicts our findings.

Additional research found that sleep disorder-related screen use was more common among females, who were more vulnerable. A Brazilian school-based study reported a link between increased screen time and lower sleep quality and duration, especially in inadequately active adolescents [37].

In large adolescent cohort studies in the UAE and Brunei, female sex and higher screen use, especially on smartphones and on weekends or in bed, were independently related to lower sleep quality [38,39]. A gender-based study suggested that adolescent females have more severe insomnia symptoms and a greater functional association of sleep problems than males [40,41,42].

Thus, while the wider evidence does support the notion that screen-insomnia connections tend to be small and that females report increased insomnia severity, the exact subtypes involved differ across studies. The revised analyses identified associations involving weekday job or internship-related use, entertainment use, and social-media use. Differences between these findings and previous studies may reflect differences in activity definitions, timing of use, and sample characteristics.

Consequently, the specific subtype associations observed here should be viewed as preliminary.

### 4.3. Postural Habits

The study showed that the adjusted postural-habit models explained only 0.6–2.1% of the variability in Q-BAPHYP scores (adjusted R^2^ = 0.006–0.021). Weekday and weekend entertainment and social-media use were associated with lower Q-BAPHYP scores after FDR adjustment. Although lower scores indicate better self-reported postural habits, these unexpected associations should not be interpreted as evidence that screen use improves posture. Residual confounding, reverse causation, measurement characteristics, and chance remain possible explanations that cannot be distinguished using these data. However, the models provided very limited explanatory information. Statistical significance does not establish clinical importance, and these cross-sectional findings do not provide a sufficient basis for practical recommendations concerning screen use and posture. A 2024 systematic review analysis of electronic screens and head/neck posture in adolescents revealed that computer use does not consistently result in postural changes and highlighted the limited, weak evidence supporting significant causal relationships between screen time and posture [43].

A scoping review (2019–2024) identified correlations between extended screen usage and musculoskeletal issues while emphasizing various contributing factors (sedentary lifestyle, ergonomics, and overall lifestyle), suggesting that screen exposure alone is a limited predictor of postural problems [11].

Previous studies have examined gender differences in posture during screen use. A previous study of Australian adolescents indicated that males exhibited increased head and neck flexion while using computers and that posture-screen connections varied by gender [44]. Another investigation, including young adults, revealed that males exhibited greater head and neck flexion angles than females while using mobile phones, with these angles correlating with the extent of computer usage, indicating poor or hazardous postural habits among males [45].

### 4.4. Health-Related Quality of Life (PedsQL)

The research showed that the adjusted PedsQL models explained 3.7–6.0% of the variability in health-related quality of life. Weekday job or internship-related use, weekday entertainment use, and weekday and weekend social-media use were associated with lower PedsQL scores after FDR adjustment. The estimated differences per additional hour/day were small, and their clinical importance remains uncertain. Physical activity, sleep habits, psychological distress, socioeconomic circumstances, and family environment were not measured; residual confounding therefore remains possible, and the observed associations should not be interpreted as causal.

The statistically significant associations represented small estimated score differences per additional hour/day; their clinical importance cannot be established from statistical significance alone. Specifically, weekday job or internship-related screen use was associated with lower PedsQL scores (B = −0.574; 95% CI, −0.967 to −0.181; FDR-adjusted *p* = 0.018). The estimated difference was small, and its clinical importance remains uncertain. However, because physical activity, sleep quality, psychosocial characteristics, and other potential confounders were not measured in the present study, these explanations remain speculative and cannot be verified with the current data. This limitation must be considered when interpreting the observed associations.

Recent research largely shows that screen time accounts for only a minor portion of the variance in adolescents’ HRQoL, while gender and broader psychosocial or lifestyle determinants are equally significant as screen exposure. A comprehensive survey conducted in Hong Kong using the PedsQL revealed a positive correlation between HRQoL and both sleep and physical activity, and a negative correlation with screen time. Other models showed that changing timing to improve sleep and physical activity while decreasing screen time could enhance HRQoL; however, the effect sizes were modest [19]. In the present study, the adjusted activity-specific PedsQL models explained 3.7–6.0% of the variability in health-related quality-of-life scores.

A UK cohort study found a link between nighttime mobile phone use and lower sleep quality and HRQoL, suggesting that screen time is a risk factor but not the main cause of overall well-being [46]. A longitudinal study from Australia revealed that adolescent females usually reported lower HRQoL than males in both physical and psychological domains, with a temporal decrease indicating a gender-specific vulnerability [47]. Also, Iranian longitudinal studies showed that males generally exhibited higher HRQoL scores than females in both mental and physical domains, highlighting persistent gender differences [48].

### 4.5. Limitations of the Study

The study used a self-selected convenience sample recruited through an online English-language questionnaire. Although participants came from multiple governorates and rural, urban, and mixed settings, this geographic diversity does not establish national representativeness. The estimated response rate of approximately 27% raises the possibility of nonresponse bias, as respondents may have differed from adolescents who did not participate. This rate is approximate because the exact recruitment denominator was not recorded.

Participation also required access to the online questionnaire and self-confirmed English comprehension, potentially underrepresenting adolescents with limited digital access or English proficiency. English proficiency was not formally assessed, and differences in comprehension may have affected responses. The findings should therefore be interpreted within this sample and cannot be assumed to represent all Egyptian adolescents.

Unsubmitted or abandoned questionnaires were not available for assessment. We therefore could not evaluate whether adolescents who did not complete the survey differed from those included, and selection related to survey completion cannot be excluded.

Physical activity, sleep hygiene, sleep duration and timing, psychological distress, socioeconomic circumstances, family environment, and academic workload were not measured. Adjustment for age, sex, BMI, educational level, and residence cannot exclude confounding by these factors. Robust standard errors, multiplicity adjustment, and sensitivity analyses address specific statistical concerns but cannot compensate for unmeasured confounding. Such confounding may influence the magnitude and direction of the observed associations and remains a central limitation of their interpretation.

Adjustment for age, sex, BMI, educational level, and residence therefore does not adequately exclude confounding by these factors. Residual confounding is a central limitation, and the reported associations should not be interpreted as effects independent of these unmeasured influences. The models accounted for only a small proportion of variation in the outcomes; the findings should therefore be interpreted cautiously. Each regression model included one screen-use measure and did not adjust for the other screen-use activities. The estimates therefore do not isolate associations specific to an activity independently of other screen exposures. Because activity durations may overlap and participants may engage in several forms of screen use, an observed association may partly reflect accompanying screen-use patterns.

Analyzing the Q-BAPHYP total score may have obscured associations specific to its individual dimensions; the component-level descriptive summaries do not resolve this limitation.

The activity-specific analytical strategy was developed during manuscript revision after the data had been examined. The resulting estimates are therefore exploratory rather than confirmatory. Although FDR adjustment addresses multiplicity across the 30 reported comparisons, it does not account for the influence of data-informed analytical decisions. These findings require evaluation in an independent sample using a prospectively specified analysis plan. The original sample-size calculation did not account for the revised set of exposure–outcome comparisons or supplementary analyses. Adequate power for each analysis, particularly for small associations, cannot therefore be assumed. Estimates that did not meet the significance threshold should not be interpreted as demonstrating the absence of an association.

Screen time and health outcomes were self-reported and may be affected by recall error or differences in how participants interpreted the questions. The questionnaire also did not distinguish phone use during teacher-led lessons from homework or other study/work-related activities, so we cannot determine how much in-class use contributed to that category. Although participants were all aged 13–18 years and age and educational level were included in the adjusted models, we did not specifically test whether associations differed across ages or educational levels.

Finally, the cross-sectional design cannot establish temporal order or causal relationships. Screen-use patterns may be associated with insomnia, postural habits, and health-related quality of life, but adolescents experiencing these difficulties may also engage in different patterns of screen use. Because exposures and outcomes were self-reported, recall error and shared reporting tendencies may also have influenced the observed associations.

## 5. Conclusions

Exploratory analyses in this self-selected Egyptian adolescent sample identified associations between activity-specific screen-use duration and the three health outcomes. The findings apply to the recruited sample and should not be generalized to the Egyptian adolescent population. After FDR adjustment, weekday job or internship-related use, weekday and weekend entertainment use, and weekday and weekend social-media use were associated with higher insomnia scores. Weekday and weekend entertainment and social-media use were associated with lower Q-BAPHYP scores, but these exploratory findings do not establish a postural benefit. Weekday job or internship-related use, weekday entertainment use, and weekday and weekend social-media use were associated with lower health-related quality-of-life scores. Study or school-related use and gaming did not meet the FDR-adjusted significance threshold for any outcome. These estimates were not mutually adjusted for other screen-use activities and do not establish independent associations for individual activities. The estimated score differences were small, and the models explained limited outcome variability. Statistical significance does not establish clinical importance, and these exploratory associations do not support clinical recommendations or causal conclusions. Future research should incorporate lifestyle, psychological, social, and environmental factors to better understand these relationships.

## Figures and Tables

**Table 1:** Demographic characteristics of participants (*n* = 530).

Characteristic	Value
Age, years	15.98 (1.50)
BMI, kg/m^2^	23.18 (3.94)
Female	265 (50.00%)
Male	265 (50.00%)
Rural residence	258 (48.68%)
Urban residence	139 (26.23%)
Mixed residence	133 (25.09%)
Primary school	14 (2.64%)
Preparatory school	126 (23.77%)
Secondary school	324 (61.13%)
University	66 (12.45%)

Continuous variables are reported as mean (SD); categorical variables are reported as n (%).

**Table 2:** Screen-use duration on weekdays and weekends (*n* = 530).

Activity	Weekday, h/day	Weekend, h/day
Study or school-related	4.00 (3.00–6.00)	4.00 (2.00–6.00)
Job or internship-related	3.00 (1.00–5.00)	2.00 (0.50–5.00)
Entertainment	3.00 (2.00–5.00)	4.00 (3.00–6.00)
Gaming	2.00 (1.00–5.00)	3.00 (2.00–6.00)
Social media	3.00 (2.00–5.00)	4.00 (3.00–7.00)

Values are median (Q1–Q3). Self-reported activity durations may overlap. Reports of 24 h/day were retained in the main analyses and examined in sensitivity analyses (Supplementary Table S7).

**Table 3:** Descriptive statistics of insomnia, postural habits, and health-related quality of life (*n* = 530).

Measure	Mean (SD)	Median (Q1–Q3)
ISI total	11.73 (4.72)	12.00 (9.00–15.00)
Q-BAPHYP total	1.26 (12.23)	1.00 (−6.00 to 8.00)
PedsQL total	65.93 (13.68)	65.22 (56.52–75.00)
Physical functioning	67.15 (16.94)	65.62 (56.25–81.25)
Emotional functioning	59.45 (18.66)	60.00 (50.00–70.00)
Social functioning	71.89 (18.55)	70.00 (60.00–85.00)
School functioning	64.51 (18.12)	65.00 (50.00–75.00)
Psychosocial summary	65.28 (14.24)	65.00 (55.00–75.00)

ISI: Insomnia Severity Index; Q-BAPHYP: Body Awareness of Postural Habits in Young People questionnaire; PedsQL: Pediatric Quality of Life Inventory.

**Table 4:** Multiple linear regression associations with ISI total score.

Activity	Period	B	95% CI	*p*	FDR-Adjusted *p*	Adjusted R^2^
Study or school-related	Weekday	0.092	−0.030 to 0.214	0.138	0.198	0.061
Study or school-related	Weekend	0.118	0.012 to 0.224	0.029	0.06	0.066
Job or internship-related	Weekday	0.218	0.109 to 0.327	<0.001	0.002	0.079
Job or internship-related	Weekend	0.113	0.003 to 0.223	0.044	0.073	0.064
Entertainment	Weekday	0.232	0.097 to 0.367	<0.001	0.005	0.079
Entertainment	Weekend	0.161	0.042 to 0.279	0.008	0.027	0.072
Gaming	Weekday	0.058	−0.080 to 0.197	0.409	0.455	0.059
Gaming	Weekend	0.079	−0.033 to 0.190	0.166	0.226	0.061
Social media	Weekday	0.176	0.077 to 0.275	<0.001	0.005	0.08
Social media	Weekend	0.209	0.106 to 0.311	<0.001	0.002	0.092

Each row represents a separate adjusted model (*n* = 530). B represents the outcome-score difference per additional hour/day. All models adjust for age, sex, residence, educational level, and continuous BMI. Confidence intervals use HC3 robust standard errors and are pointwise rather than multiplicity-adjusted. FDR-adjusted *p* -values account for all 30 screen-use associations.

**Table 5:** Multiple linear regression associations with Q-BAPHYP total score.

Activity	Period	B	95% CI	*p*	FDR-Adjusted *p*	Adjusted R^2^
Study or school-related	Weekday	−0.305	−0.585 to −0.025	0.033	0.06	0.013
Study or school-related	Weekend	−0.082	−0.378 to 0.214	0.587	0.607	0.007
Job or internship-related	Weekday	−0.106	−0.388 to 0.175	0.458	0.491	0.007
Job or internship-related	Weekend	0.067	−0.192 to 0.327	0.611	0.611	0.006
Entertainment	Weekday	−0.414	−0.731 to −0.098	0.01	0.03	0.016
Entertainment	Weekend	−0.413	−0.730 to −0.095	0.011	0.03	0.021
Gaming	Weekday	−0.304	−0.585 to −0.023	0.034	0.06	0.013
Gaming	Weekend	−0.168	−0.427 to 0.091	0.204	0.266	0.008
Social media	Weekday	−0.338	−0.537 to −0.139	<0.001	0.005	0.018
Social media	Weekend	−0.254	−0.460 to −0.047	0.016	0.037	0.014

Each row represents a separate adjusted model (N = 530). B represents the outcome-score difference per additional hour/day. All models adjust for age, sex, residence, educational level, and continuous BMI. Confidence intervals use HC3 robust standard errors and are pointwise rather than multiplicity-adjusted. FDR-adjusted *p* values account for all 30 screen-use associations.

**Table 6:** Multiple linear regression associations with PedsQL total score.

Activity	Period	B	95% CI	*p*	FDR-Adjusted *p*	Adjusted R^2^
Study or school-related	Weekday	−0.206	−0.556 to 0.143	0.247	0.296	0.038
Study or school-related	Weekend	−0.148	−0.465 to 0.169	0.36	0.416	0.037
Job or internship-related	Weekday	−0.574	−0.967 to −0.181	0.004	0.018	0.054
Job or internship-related	Weekend	−0.378	−0.775 to 0.019	0.062	0.093	0.044
Entertainment	Weekday	−0.562	−1.007 to −0.116	0.014	0.034	0.051
Entertainment	Weekend	−0.417	−0.801 to −0.033	0.033	0.06	0.048
Gaming	Weekday	−0.285	−0.759 to 0.189	0.238	0.296	0.04
Gaming	Weekend	−0.376	−0.751 to −0.002	0.049	0.077	0.046
Social media	Weekday	−0.471	−0.815 to −0.127	0.007	0.027	0.055
Social media	Weekend	−0.506	−0.839 to −0.174	0.003	0.014	0.06

Each row represents a separate adjusted model (*n* = 530). B represents the outcome-score difference per additional hour/day. All models adjust for age, sex, residence, educational level, and continuous BMI. Confidence intervals use HC3 robust standard errors and are pointwise rather than multiplicity-adjusted. FDR-adjusted *p* -values account for all 30 screen-use associations.

## Data Availability

The raw data supporting the conclusions of this article will be made available by the authors on request.

## Supplementary Materials

The following supporting information can be downloaded at: https://www.mdpi.com/article/10.3390/healthcare14203498/s1, Table S1: Study/school-related screen-use classifications. Descriptive frequencies of the recorded typical, balanced, and excessive categories; these classifications were not used in the regression analyses. Table S2: Screen-use duration by age and educational level. Weekday and weekend activity-specific durations summarized using means, standard deviations, medians, and interquartile ranges. Table S3: Descriptive statistics for Q-BAPHYP components. Component-level means, standard deviations, medians, quartiles, and observed ranges. Table S4: Alternative outcome analyses for all 30 screen-use activity–outcome comparisons. Adjusted binary logistic regression for moderate or severe insomnia (ISI ≥15) and median quantile regression for Q-BAPHYP and PedsQL, with corresponding linear-regression estimates. Table S5: Functional-form comparisons for all 30 screen-use activity–outcome models. Exploratory comparisons of linear and spline specifications, including model R^2^ values and likelihood-ratio tests for added nonlinearity. Table S6: Sensitivity of activity-specific regression estimates to influential observations. Comparison of main-model estimates with estimates obtained after excluding observations with Cook’s distance >4/530. Table S7: Sensitivity of activity-specific regression estimates to self-reported 24-hour screen-use values. Comparison of main-model estimates with estimates obtained after replacing reported 24-h values with 20 h/day as a sensitivity scenario. Figure S1: Residual-versus-fitted and normal Q–Q plots for adjusted ISI models: weekday and weekend study/school-related use, weekday and weekend job/internship-related use, and weekday entertainment use. Figure S2: Residual-versus-fitted and normal Q–Q plots for adjusted ISI models: weekend entertainment use, weekday and weekend gaming, and weekday and weekend social-media use. Figure S3: Residual-versus-fitted and normal Q–Q plots for adjusted Q-BAPHYP models: weekday and weekend study/school-related use, weekday and weekend job/internship-related use, and weekday entertainment use. Figure S4: Residual-versus-fitted and normal Q–Q plots for adjusted Q-BAPHYP models: weekend entertainment use, weekday and weekend gaming, and weekday and weekend social-media use. Figure S5: Residual-versus-fitted and normal Q–Q plots for adjusted PedsQL models: weekday and weekend study/school-related use, weekday and weekend job/internship-related use, and weekday entertainment use. Figure S6: Residual-versus-fitted and normal Q–Q plots for adjusted PedsQL models: weekend entertainment use, weekday and weekend gaming, and weekday and weekend social-media use. For Figures S1–S6, each model includes 530 participants and one continuous screen-use measure, adjusted for age, sex, residence, educational level, and continuous BMI. Left panels show raw residuals against fitted scores; right panels show residuals divided by the model residual standard deviation against theoretical standard-normal quantiles.

## Abbreviations

The following abbreviations are used in this manuscript:

BMI	Body Mass Index
Gen Z	Generation Z
HRQoL	Health-related quality of life
ISI	Insomnia Severity Index
MENA	Middle East and North Africa
PedsQL	Pediatric Quality of Life Inventory
Q-BAPHYP	The Questionnaire on Body Awareness of Postural Habits in Young People
QueST	The Questionnaire for Screen Time of Adolescents
STROBE	Strengthening the Reporting of Observational Studies in Epidemiology
