# Supplementary material for: Screen Culture’s Association with Generation Z Adolescents’ Posture and Well-Being in Egypt: Cross-Sectional Study
Source: Healthcare (Basel). 2026 Oct 18;14(20):3498. doi: 10.3390/healthcare14203498 (PMC13704529; doi:10.3390/healthcare14203498)

Supplementary Figure S1. ISI total — models 1–5
N = 530 per model; one activity exposure + age, sex, residence, education and BMI

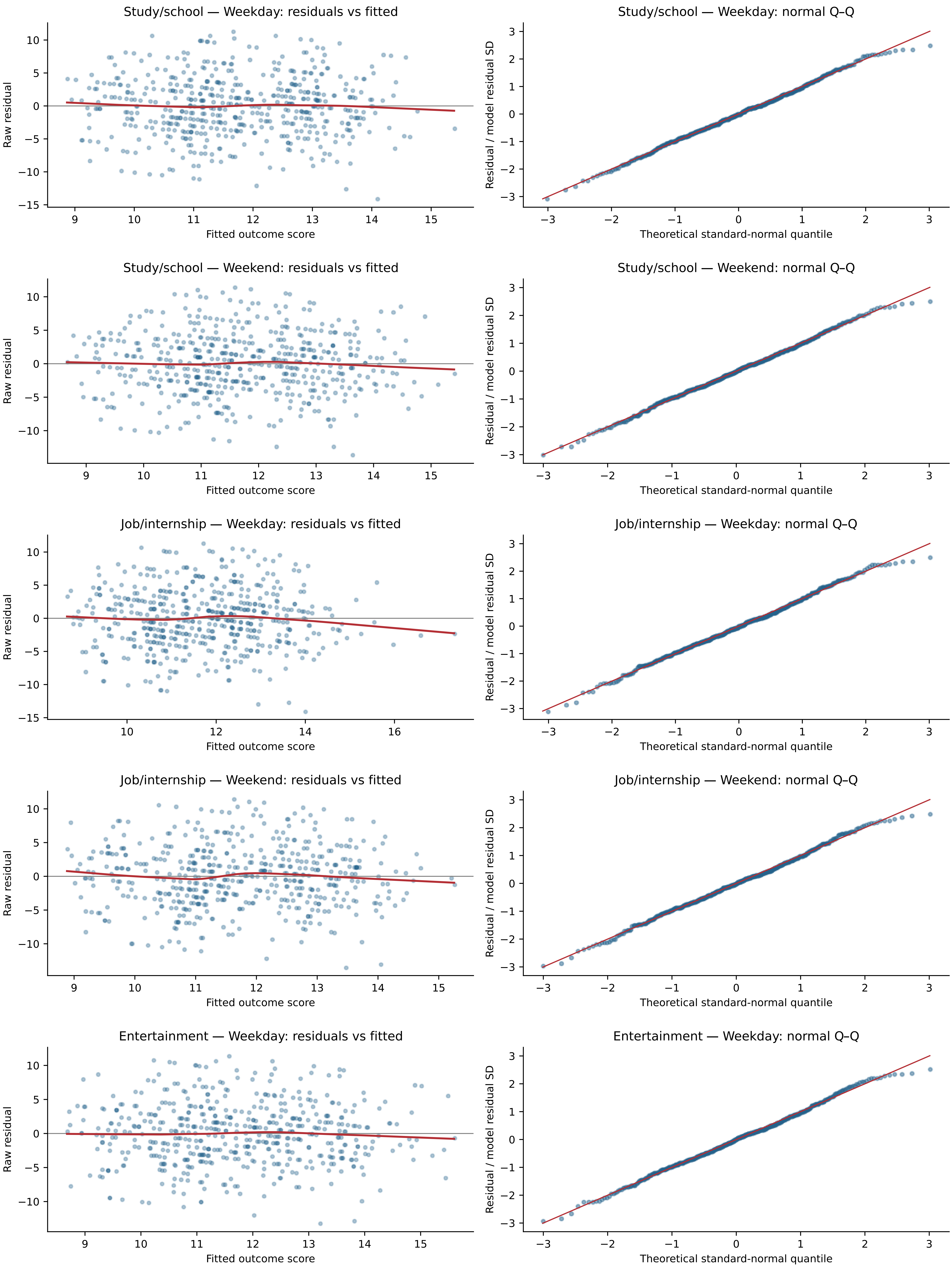

Supplementary Figure S2. ISI total — models 6–10
N = 530 per model; one activity exposure + age, sex, residence, education and BMI

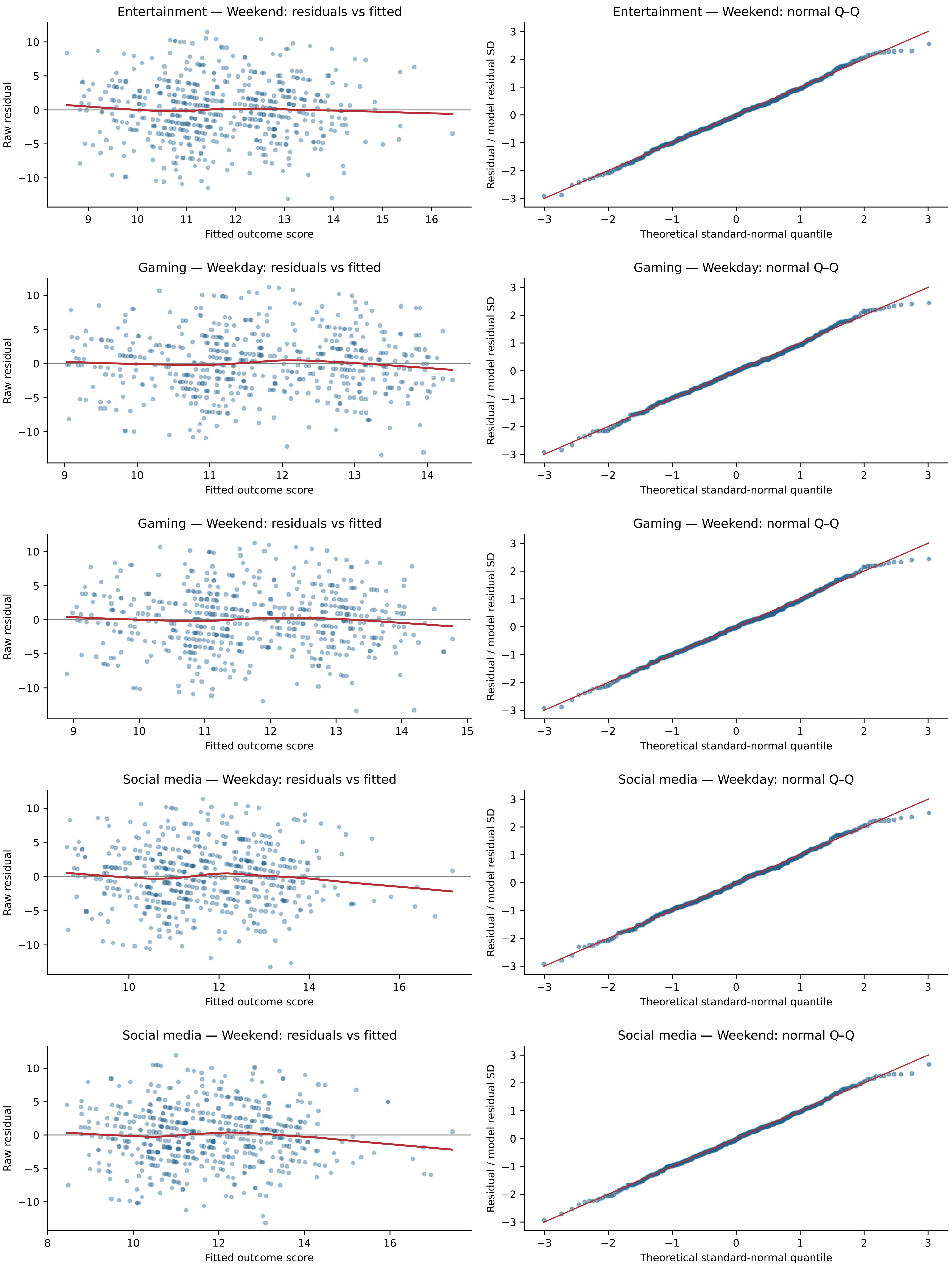

Supplementary Figure S3. Q-BAPHYP total — models 1–5
N = 530 per model; one activity exposure + age, sex, residence, education and BMI

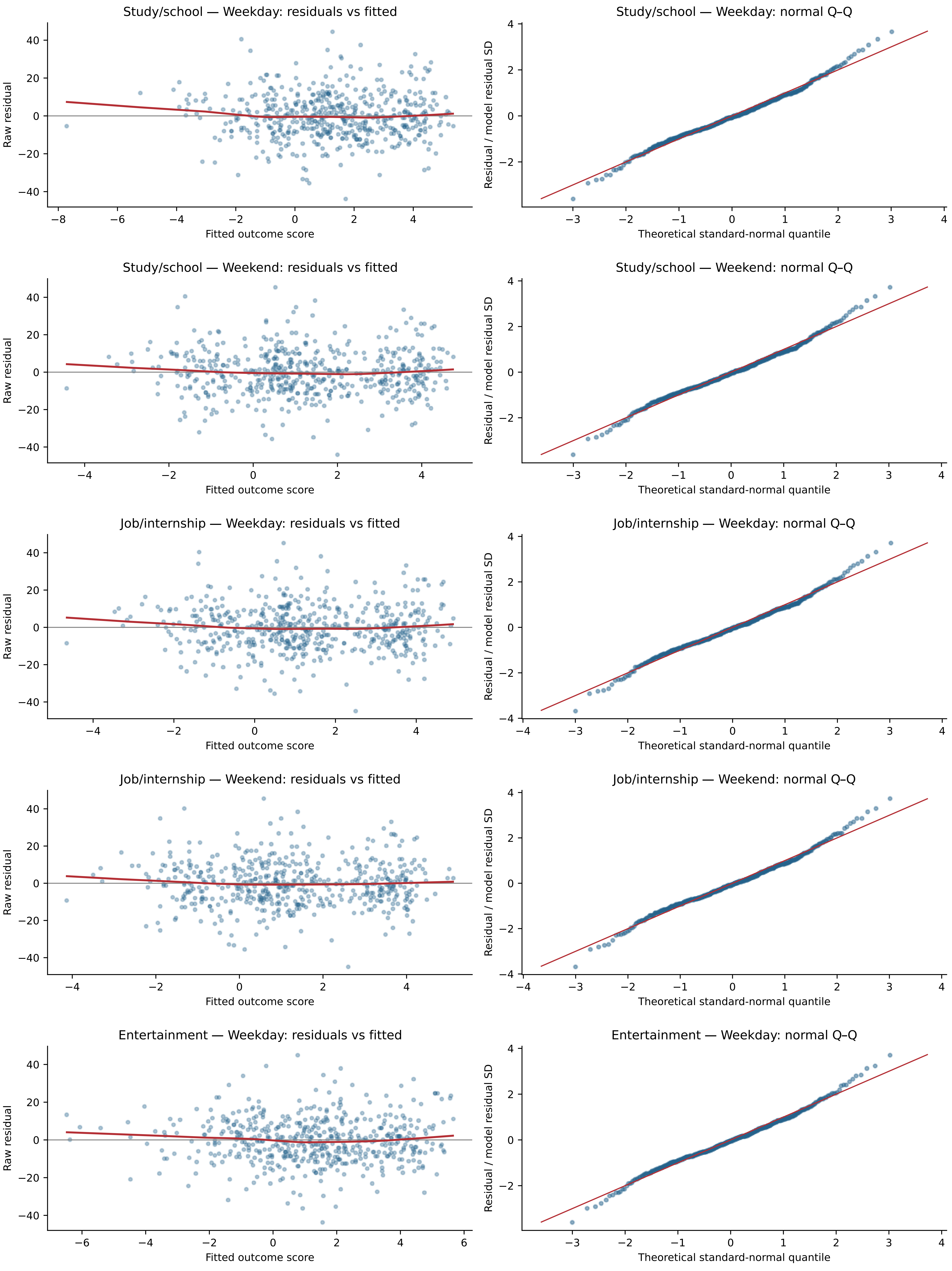

Supplementary Figure S4. Q-BAPHYP total — models 6–10
N = 530 per model; one activity exposure + age, sex, residence, education and BMI

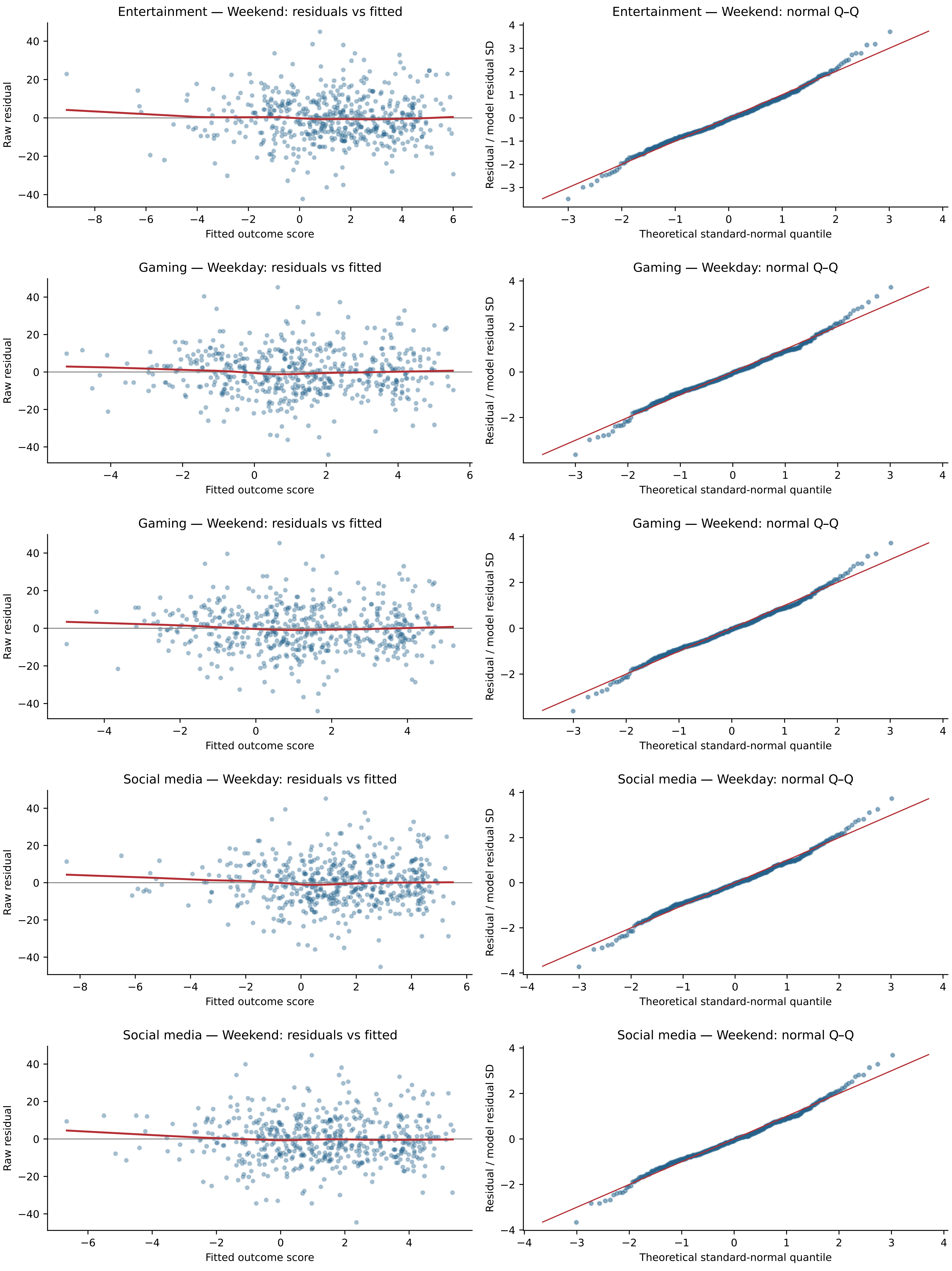

Supplementary Figure S5. PedsQL total — models 1–5
N = 530 per model; one activity exposure + age, sex, residence, education and BMI

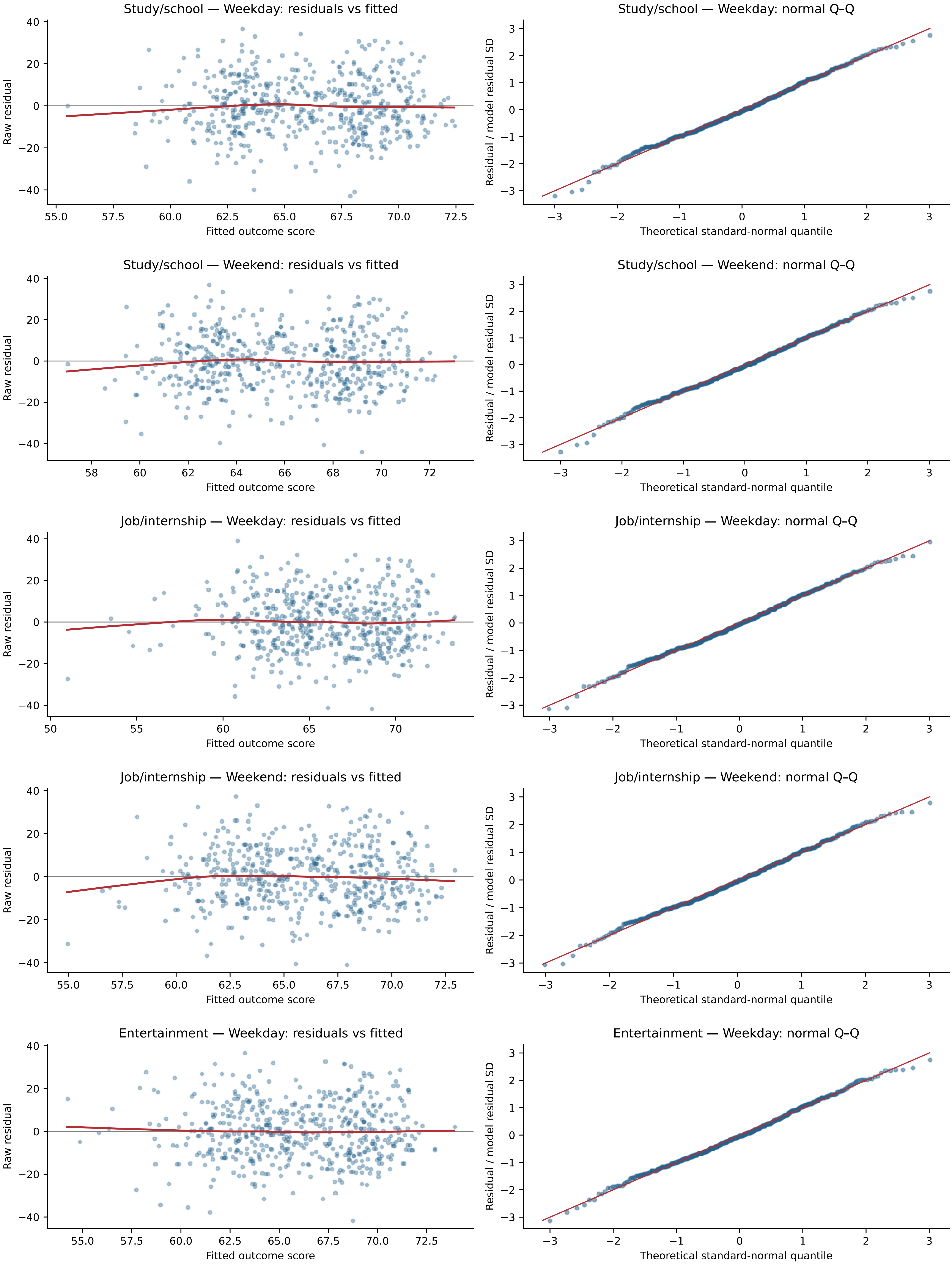

Supplementary Figure S6. PedsQL total — models 6–10
N = 530 per model; one activity exposure + age, sex, residence, education and BMI

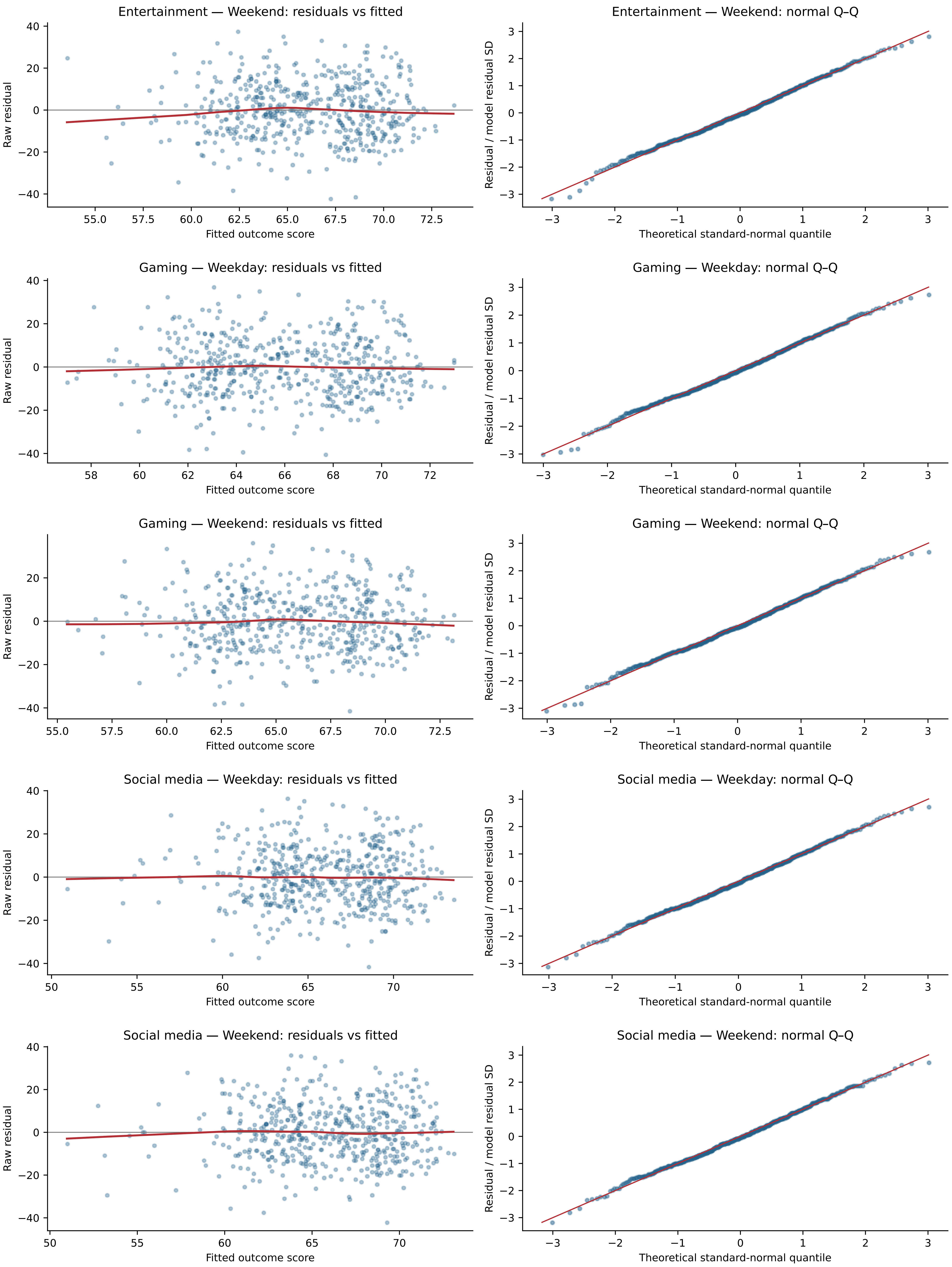

Supplement: Supplementary file 1 [file healthcare-14-03498-s001.zip › Supplementary Figures S1–S6.pdf]
