# Supplementary material for: Screen Culture’s Association with Generation Z Adolescents’ Posture and Well-Being in Egypt: Cross-Sectional Study
Source: Healthcare (Basel). 2026 Oct 18;14(20):3498. doi: 10.3390/healthcare14203498 (PMC13704529; doi:10.3390/healthcare14203498)
Supplement: Supplementary file 1 [file healthcare-14-03498-s001.zip › Supplementary Table S1.pdf]

**Supplementary Table S1.** Study/school-related screen-use classifications

| Period | Classification | n | % |
|---|---|---|---|
| **Weekday** | Typical | 117 | 22.08 |
| **Weekday** | Balanced | 87 | 16.42 |
| **Weekday** | Excessive | 326 | 61.51 |
| **Weekend** | Typical | 140 | 26.42 |
| **Weekend** | Balanced | 104 | 19.62 |
| **Weekend** | Excessive | 286 | 53.96 |

N = 530. These classifications reproduce the labels in the analysed dataset and were not included in the revised regression models. Weekday reports of exactly 2 h/day were assigned inconsistently: 29 were labelled typical and 45 balanced. Accordingly, this table should not be interpreted as establishing a consistent guideline-based classification.
