# Supplementary material for: Screen Culture’s Association with Generation Z Adolescents’ Posture and Well-Being in Egypt: Cross-Sectional Study
Source: Healthcare (Basel). 2026 Oct 18;14(20):3498. doi: 10.3390/healthcare14203498 (PMC13704529; doi:10.3390/healthcare14203498)
Supplement: Supplementary file 1 [file healthcare-14-03498-s001.zip › Supplementary Table S2.pdf]

Supplementary Table S2. Screen-Use Duration by Age and Educational Level

Group	n	Activity	Weekday mean (SD)	Weekday median (Q1–Q3)	Weekend mean (SD)	Weekend median (Q1–Q3)
**Age 13 years**	45	Study/school	4.244 (2.522)	4.000 (2.000–6.000)	5.033 (4.416)	4.000 (3.000–6.000)
**Age 13 years**	45	Job/internship	2.422 (2.259)	2.000 (0.500–3.000)	2.922 (2.981)	2.000 (0.000–4.000)
**Age 13 years**	45	Entertainment	3.700 (2.079)	3.000 (2.000–5.000)	4.789 (3.328)	4.000 (3.000–6.000)
**Age 13 years**	45	Gaming	3.867 (2.966)	3.000 (2.000–5.000)	4.822 (4.345)	4.000 (2.000–5.000)
**Age 13 years**	45	Social media	3.711 (3.465)	2.000 (1.000–5.000)	3.822 (2.326)	4.000 (2.000–5.000)
**Age 14 years**	48	Study/school	4.438 (3.175)	4.000 (2.000–6.000)	4.115 (3.067)	4.000 (2.000–6.000)
**Age 14 years**	48	Job/internship	2.854 (2.703)	2.000 (1.000–4.000)	2.625 (3.015)	2.000 (0.000–3.250)
**Age 14 years**	48	Entertainment	3.344 (2.622)	3.000 (2.000–4.000)	3.802 (2.940)	3.000 (2.000–5.000)
**Age 14 years**	48	Gaming	3.688 (3.230)	3.000 (1.750–5.000)	3.823 (2.996)	3.000 (2.000–5.000)
**Age 14 years**	48	Social media	3.865 (3.295)	3.000 (2.000–5.000)	4.312 (2.933)	4.000 (2.000–5.000)
**Age 15 years**	93	Study/school	4.640 (3.006)	4.000 (3.000–6.000)	4.565 (4.189)	4.000 (2.000–6.000)
**Age 15 years**	93	Job/internship	3.145 (3.243)	3.000 (1.000–4.000)	3.108 (3.175)	2.000 (0.500–5.000)
**Age 15 years**	93	Entertainment	3.629 (3.014)	3.000 (2.000–5.000)	5.054 (3.683)	4.000 (3.000–6.000)
**Age 15 years**	93	Gaming	3.532 (3.725)	3.000 (1.000–5.000)	4.548 (3.954)	4.000 (2.000–6.000)
**Age 15 years**	93	Social media	4.398 (3.906)	3.000 (2.000–6.000)	4.968 (3.706)	4.000 (2.000–7.000)
**Age 16 years**	130	Study/school	4.892 (3.477)	4.000 (3.000–6.000)	4.485 (3.619)	4.000 (2.000–6.000)
**Age 16 years**	130	Job/internship	3.250 (3.701)	2.000 (1.000–4.000)	3.342 (4.186)	2.000 (0.500–5.000)
**Age 16 years**	130	Entertainment	4.169 (3.997)	3.000 (2.000–5.000)	4.908 (4.052)	4.000 (3.000–6.000)
**Age 16 years**	130	Gaming	3.319 (3.809)	2.000 (1.000–4.750)	4.435 (3.928)	3.500 (2.000–6.000)
**Age 16 years**	130	Social media	4.708 (4.952)	3.000 (2.000–6.000)	6.135 (5.309)	5.000 (3.000–8.000)
**Age 17 years**	116	Study/school	4.797 (3.010)	4.000 (3.000–6.000)	4.478 (3.495)	4.000 (2.000–6.000)
**Age 17 years**	116	Job/internship	3.431 (3.497)	3.000 (1.000–5.000)	3.276 (3.450)	2.500 (0.875–5.000)
**Age 17 years**	116	Entertainment	3.569 (2.762)	3.000 (2.000–5.000)	4.560 (3.517)	4.000 (2.000–6.000)
**Age 17 years**	116	Gaming	2.884 (2.902)	2.000 (1.000–4.000)	3.629 (3.216)	3.000 (2.000–5.000)
**Age 17 years**	116	Social media	3.677 (2.476)	3.000 (2.000–5.000)	5.228 (3.579)	5.000 (3.000–7.000)
**Age 18 years**	98	Study/school	5.816 (4.065)	4.500 (3.000–7.750)	4.964 (4.405)	4.000 (2.000–6.000)
**Age 18 years**	98	Job/internship	3.745 (2.967)	4.000 (2.000–5.000)	3.429 (3.024)	3.000 (1.000–5.000)
**Age 18 years**	98	Entertainment	3.633 (2.414)	3.000 (2.000–5.000)	5.316 (3.591)	4.000 (3.000–7.000)
**Age 18 years**	98	Gaming	3.209 (3.195)	2.500 (1.000–4.000)	4.622 (3.901)	4.000 (2.000–6.000)
**Age 18 years**	98	Social media	4.811 (4.924)	4.000 (2.000–5.000)	6.291 (4.949)	5.000 (3.000–8.000)
**Primary school**	14	Study/school	4.107 (2.761)	3.500 (2.000–5.750)	5.893 (5.871)	4.000 (3.000–7.000)
**Primary school**	14	Job/internship	2.214 (1.805)	1.500 (1.000–3.000)	4.571 (3.275)	4.500 (2.250–6.750)
**Primary school**	14	Entertainment	2.821 (2.569)	2.000 (1.250–3.000)	5.429 (5.996)	3.000 (3.000–5.000)
**Primary school**	14	Gaming	6.786 (6.266)	5.000 (2.250–8.000)	5.714 (5.497)	4.000 (2.000–7.250)
**Primary school**	14	Social media	5.464 (5.753)	3.000 (2.000–6.000)	4.821 (4.259)	5.000 (2.250–5.750)
**Preparatory school**	126	Study/school	4.321 (2.652)	4.000 (2.000–6.000)	4.504 (3.423)	4.000 (2.000–6.000)
**Preparatory school**	126	Job/internship	3.052 (3.036)	3.000 (1.000–4.000)	2.782 (3.185)	2.000 (0.000–4.000)
**Preparatory school**	126	Entertainment	3.825 (3.162)	3.000 (2.000–5.000)	4.829 (3.355)	4.000 (3.000–6.000)

Group	n	Activity	Weekday mean (SD)	Weekday median (Q1–Q3)	Weekend mean (SD)	Weekend median (Q1–Q3)
**Preparatory school**	126	Gaming	3.675 (3.447)	3.000 (1.000–5.000)	4.679 (4.198)	4.000 (2.000–6.000)
**Preparatory school**	126	Social media	4.238 (3.997)	3.000 (2.000–5.000)	4.869 (3.713)	4.000 (2.250–6.000)
**Secondary school**	324	Study/school	4.923 (3.429)	4.000 (3.000–6.000)	4.404 (3.796)	4.000 (2.000–6.000)
**Secondary school**	324	Job/internship	3.276 (3.395)	2.000 (1.000–5.000)	3.216 (3.579)	2.000 (1.000–5.000)
**Secondary school**	324	Entertainment	3.694 (3.095)	3.000 (2.000–5.000)	4.563 (3.514)	4.000 (2.000–6.000)
**Secondary school**	324	Gaming	3.068 (3.180)	2.000 (1.000–4.000)	4.011 (3.462)	3.000 (2.000–5.000)
**Secondary school**	324	Social media	4.123 (3.851)	3.000 (2.000–5.000)	5.448 (4.373)	4.000 (3.000–7.000)
**University**	66	Study/school	6.076 (3.904)	5.000 (3.250–7.000)	5.462 (4.428)	5.000 (2.250–7.000)
**University**	66	Job/internship	3.773 (3.222)	3.000 (1.250–5.000)	3.644 (3.242)	3.500 (1.000–6.000)
**University**	66	Entertainment	3.909 (2.615)	3.500 (2.000–5.000)	5.955 (4.012)	5.500 (3.000–8.000)
**University**	66	Gaming	3.152 (2.944)	2.000 (1.000–5.000)	4.621 (3.707)	4.000 (2.000–7.000)
**University**	66	Social media	4.924 (4.816)	4.000 (2.000–6.000)	6.295 (4.802)	5.000 (3.000–8.000)

All durations are hours/day. Weekday and weekend summaries are displayed side by side for each activity. These are descriptive subgroup summaries; no between-group or interaction tests are reported.
