# Supplementary material for: Screen Culture’s Association with Generation Z Adolescents’ Posture and Well-Being in Egypt: Cross-Sectional Study
Source: Healthcare (Basel). 2026 Oct 18;14(20):3498. doi: 10.3390/healthcare14203498 (PMC13704529; doi:10.3390/healthcare14203498)
Supplement: Supplementary file 1 [file healthcare-14-03498-s001.zip › Supplementary Table S3.pdf]

**Supplementary Table S3.** Descriptive Statistics for Q-BAPHYP Components

| Component | Mean | SD | Median | Q1 | Q3 | Minimum | Maximum |
|---|---|---|---|---|---|---|---|
| **Sitting in classroom** | 1.057 | 4.840 | 1.000 | −2.000 | 4.000 | −13.000 | 14.000 |
| **Standing in classroom** | 0.815 | 2.052 | 1.000 | 0.000 | 2.000 | −4.000 | 4.000 |
| **Movement in classroom** | −0.102 | 1.329 | 0.000 | −1.000 | 1.000 | −2.000 | 2.000 |
| **Sitting at home** | 0.670 | 4.752 | 1.000 | −2.000 | 4.000 | −15.000 | 15.000 |
| **Lying down at home** | 0.394 | 2.195 | 0.000 | −1.000 | 2.000 | −5.000 | 6.000 |
| **Movement at home** | 0.066 | 1.339 | 0.000 | −1.000 | 1.000 | −2.000 | 2.000 |
| **Standing at home** | 0.662 | 2.017 | 0.000 | 0.000 | 2.000 | −4.000 | 4.000 |
| **TV/computer posture** | −0.838 | 3.994 | 0.000 | −4.000 | 3.000 | −6.000 | 6.000 |
| **Carrying a backpack** | 0.191 | 2.236 | 0.000 | −2.000 | 2.000 | −4.000 | 4.000 |
| **Picking up an object** | −0.015 | 2.001 | 0.000 | −1.000 | 1.000 | −4.000 | 4.000 |
| **Teacher-related score** | −1.640 | 2.698 | −2.000 | −4.000 | 0.000 | −6.000 | 5.000 |

**N.B.** Each component had 530 observations and no missing component scores. Q1 and Q3 denote the first and third quartiles. These are component-level descriptive summaries, not separate analyses of the questionnaire's four dimensions.
