# Supplementary material for: Screen Culture’s Association with Generation Z Adolescents’ Posture and Well-Being in Egypt: Cross-Sectional Study
Source: Healthcare (Basel). 2026 Oct 18;14(20):3498. doi: 10.3390/healthcare14203498 (PMC13704529; doi:10.3390/healthcare14203498)
Supplement: Supplementary file 1 [file healthcare-14-03498-s001.zip › Supplementary Table S4.pdf]

Supplementary Table S4. Alternative Outcome Analyses for All 30 Screen-Use Activity–Outcome Comparisons

Outcome	Activity	Period	Alternative estimate	95% CI	p	Corresponding OLS B	Direction agreement
**ISI ≥15**	Study/school	Weekday	1.014	0.957 to 1.074	0.636	0.092	Same
**ISI ≥15**	Study/school	Weekend	1.046	0.995 to 1.098	0.075	0.118	Same
**ISI ≥15**	Job/internship	Weekday	1.065	1.005 to 1.130	0.034	0.218	Same
**ISI ≥15**	Job/internship	Weekend	1.054	0.997 to 1.114	0.065	0.113	Same
**ISI ≥15**	Entertainment	Weekday	1.059	0.995 to 1.128	0.073	0.232	Same
**ISI ≥15**	Entertainment	Weekend	1.054	1.000064 to 1.111	0.0497	0.161	Same
**ISI ≥15**	Gaming	Weekday	0.991	0.931 to 1.055	0.783	0.058	Different
**ISI ≥15**	Gaming	Weekend	1.01	0.957 to 1.066	0.724	0.079	Same
**ISI ≥15**	Social media	Weekday	1.047	0.999611 to 1.097	0.052	0.176	Same
**ISI ≥15**	Social media	Weekend	1.064	1.018 to 1.112	0.006	0.209	Same
**Q-BAPHYP total**	Study/school	Weekday	−0.309	−0.639 to 0.107	0.168	−0.305	Same
**Q-BAPHYP total**	Study/school	Weekend	0.009	−0.396 to 0.265	0.924	−0.082	Different
**Q-BAPHYP total**	Job/internship	Weekday	−0.060	−0.333 to 0.339	0.956	−0.106	Same
**Q-BAPHYP total**	Job/internship	Weekend	0.278	−0.166 to 0.464	0.228	0.067	Same
**Q-BAPHYP total**	Entertainment	Weekday	−0.103	−0.663 to 0.231	0.3	−0.414	Same
**Q-BAPHYP total**	Entertainment	Weekend	−0.199	−0.773 to 0.201	0.276	−0.413	Same
**Q-BAPHYP total**	Gaming	Weekday	−0.087	−0.615 to 0.189	0.376	−0.304	Same
**Q-BAPHYP total**	Gaming	Weekend	−0.022	−0.237 to 0.332	0.896	−0.168	Same
**Q-BAPHYP total**	Social media	Weekday	−0.321	−0.544 to −0.017	0.032	−0.338	Same
**Q-BAPHYP total**	Social media	Weekend	−0.116	−0.400 to 0.108	0.2	−0.254	Same
**PedsQL total**	Study/school	Weekday	−0.180	−0.480 to 0.372	0.468	−0.206	Same
**PedsQL total**	Study/school	Weekend	0.068	−0.455 to 0.374	0.904	−0.148	Different
**PedsQL total**	Job/internship	Weekday	−0.212	−0.884 to 0.089	0.184	−0.574	Same
**PedsQL total**	Job/internship	Weekend	−0.084	−0.547 to 0.244	0.544	−0.378	Same
**PedsQL total**	Entertainment	Weekday	−0.436	−0.807 to −0.001	0.044	−0.562	Same
**PedsQL total**	Entertainment	Weekend	−0.350	−0.805 to 0.088	0.092	−0.417	Same
**PedsQL total**	Gaming	Weekday	−0.237	−0.692 to 0.268	0.32	−0.285	Same
**PedsQL total**	Gaming	Weekend	−0.350	−0.651 to 0.038	0.06	−0.376	Same
**PedsQL total**	Social media	Weekday	−0.444	−0.784 to −0.026	0.028	−0.471	Same
**PedsQL total**	Social media	Weekend	−0.375	−0.834 to −0.042	0.036	−0.506	Same

Each row represents a separate model with N = 530, adjusted for age, sex, residence, educational level, and continuous BMI. ISI estimates are adjusted odds ratios for ISI ≥15 versus <15 per additional hour/day. Q-BAPHYP and PedsQL estimates are adjusted median-score differences per additional hour/day from median quantile regression. Quantile confidence intervals are percentile bootstrap intervals based on 500 resamples per model; the supplied output reports 500 successful and zero failed resamples for each quantile model. Quantile p values are the supplied exploratory bootstrap sign-tail p values. Alternative-model p values are not adjusted for multiplicity. Additional decimal places are retained for two ISI confidence limits close to 1. Direction agreement alone does not establish agreement in statistical evidence.
