# Supplementary material for: Screen Culture’s Association with Generation Z Adolescents’ Posture and Well-Being in Egypt: Cross-Sectional Study
Source: Healthcare (Basel). 2026 Oct 18;14(20):3498. doi: 10.3390/healthcare14203498 (PMC13704529; doi:10.3390/healthcare14203498)
Supplement: Supplementary file 1 [file healthcare-14-03498-s001.zip › Supplementary Table S5.pdf]

Supplementary Table S5. Functional-Form Comparisons for All 30 Screen-Use Activity–Outcome Models

Outcome	Activity	Period	Linear R^2	Spline R^2	LR χ^2	p for added nonlinearity
**ISI total**	Study/school	Weekday	0.077	0.077	0.214	0.644
**ISI total**	Study/school	Weekend	0.082	0.084	0.915	0.339
**ISI total**	Job/internship	Weekday	0.095	0.095	0.002	0.966
**ISI total**	Job/internship	Weekend	0.08	0.082	1.526	0.217
**ISI total**	Entertainment	Weekday	0.095	0.095	0.135	0.714
**ISI total**	Entertainment	Weekend	0.088	0.092	2.287	0.13
**ISI total**	Gaming	Weekday	0.075	0.075	0.479	0.489
**ISI total**	Gaming	Weekend	0.077	0.077	0.203	0.652
**ISI total**	Social media	Weekday	0.096	0.098	1.186	0.276
**ISI total**	Social media	Weekend	0.107	0.113	3.569	0.059
**Q-BAPHYP total**	Study/school	Weekday	0.029	0.03	0.393	0.531
**Q-BAPHYP total**	Study/school	Weekend	0.023	0.029	3.241	0.072
**Q-BAPHYP total**	Job/internship	Weekday	0.024	0.029	3.024	0.082
**Q-BAPHYP total**	Job/internship	Weekend	0.023	0.026	1.547	0.214
**Q-BAPHYP total**	Entertainment	Weekday	0.033	0.041	4.418	0.036
**Q-BAPHYP total**	Entertainment	Weekend	0.038	0.059	12.071	<0.001
**Q-BAPHYP total**	Gaming	Weekday	0.029	0.031	0.815	0.367
**Q-BAPHYP total**	Gaming	Weekend	0.025	0.026	0.474	0.491
**Q-BAPHYP total**	Social media	Weekday	0.035	0.039	2.353	0.125
**Q-BAPHYP total**	Social media	Weekend	0.03	0.035	2.34	0.126
**PedsQL total**	Study/school	Weekday	0.054	0.06	3.269	0.071
**PedsQL total**	Study/school	Weekend	0.054	0.054	0.028	0.867
**PedsQL total**	Job/internship	Weekday	0.07	0.07	0.079	0.778
**PedsQL total**	Job/internship	Weekend	0.061	0.074	7.563	0.006
**PedsQL total**	Entertainment	Weekday	0.067	0.067	0.268	0.604
**PedsQL total**	Entertainment	Weekend	0.064	0.069	2.629	0.105
**PedsQL total**	Gaming	Weekday	0.057	0.057	0.075	0.784
**PedsQL total**	Gaming	Weekend	0.062	0.062	0.057	0.811
**PedsQL total**	Social media	Weekday	0.071	0.072	0.595	0.441
**PedsQL total**	Social media	Weekend	0.076	0.076	0.005	0.946

N = 530 for every comparison. Models adjust for age, sex, residence, educational level, and continuous BMI. LR denotes likelihood ratio. Each comparison has one additional degree of freedom for the spline specification relative to the linear specification. These are exploratory, unadjusted ordinary OLS likelihood-ratio comparisons. A non-significant result does not establish that a relationship is strictly linear.
