# Supplementary material for: Screen Culture’s Association with Generation Z Adolescents’ Posture and Well-Being in Egypt: Cross-Sectional Study
Source: Healthcare (Basel). 2026 Oct 18;14(20):3498. doi: 10.3390/healthcare14203498 (PMC13704529; doi:10.3390/healthcare14203498)
Supplement: Supplementary file 1 [file healthcare-14-03498-s001.zip › Supplementary Table S6.pdf]

Supplementary Table S6. Sensitivity of Activity-Specific Regression Estimates to Influential Observations

Outcome	Activity	Period	Flagged n	Maximum Cook's D	Main B (95% CI)	B after exclusion (95% CI)	p after exclusion	N after exclusion
**ISI total**	Study/school	Weekday	28	0.033	0.092 (−0.030 to 0.214)	0.034 (−0.071 to 0.139)	0.523	502
**ISI total**	Study/school	Weekend	29	0.033	0.118 (0.012 to 0.224)	0.060 (−0.028 to 0.148)	0.181	501
**ISI total**	Job/internship	Weekday	29	0.033	0.218 (0.109 to 0.327)	0.167 (0.074 to 0.260)	<0.001	501
**ISI total**	Job/internship	Weekend	30	0.037	0.113 (0.003 to 0.223)	0.104 (0.013 to 0.195)	0.026	500
**ISI total**	Entertainment	Weekday	27	0.035	0.232 (0.097 to 0.367)	0.209 (0.089 to 0.328)	0.001	503
**ISI total**	Entertainment	Weekend	29	0.063	0.161 (0.042 to 0.279)	0.158 (0.058 to 0.258)	0.002	501
**ISI total**	Gaming	Weekday	29	0.054	0.058 (−0.080 to 0.197)	0.049 (−0.054 to 0.152)	0.347	501
**ISI total**	Gaming	Weekend	27	0.039	0.079 (−0.033 to 0.190)	0.090 (0.001 to 0.179)	0.049	503
**ISI total**	Social media	Weekday	25	0.035	0.176 (0.077 to 0.275)	0.154 (0.066 to 0.242)	0.001	505
**ISI total**	Social media	Weekend	32	0.042	0.209 (0.106 to 0.311)	0.230 (0.137 to 0.324)	<0.001	498
**Q-BAPHYP total**	Study/school	Weekday	26	0.034	−0.305 (−0.585 to −0.025)	−0.165 (−0.415 to 0.086)	0.197	504
**Q-BAPHYP total**	Study/school	Weekend	27	0.038	−0.082 (−0.378 to 0.214)	0.015 (−0.211 to 0.241)	0.896	503
**Q-BAPHYP total**	Job/internship	Weekday	25	0.034	−0.106 (−0.388 to 0.175)	−0.124 (−0.386 to 0.137)	0.35	505
**Q-BAPHYP total**	Job/internship	Weekend	26	0.035	0.067 (−0.192 to 0.327)	−0.020 (−0.266 to 0.225)	0.87	504
**Q-BAPHYP total**	Entertainment	Weekday	25	0.034	−0.414 (−0.731 to −0.098)	−0.313 (−0.597 to −0.029)	0.031	505
**Q-BAPHYP total**	Entertainment	Weekend	29	0.037	−0.413 (−0.730 to −0.095)	−0.390 (−0.649 to −0.131)	0.003	501
**Q-BAPHYP total**	Gaming	Weekday	25	0.033	−0.304 (−0.585 to −0.023)	−0.288 (−0.539 to −0.037)	0.024	505
**Q-BAPHYP total**	Gaming	Weekend	29	0.037	−0.168 (−0.427 to 0.091)	−0.058 (−0.274 to 0.157)	0.596	501
**Q-BAPHYP total**	Social media	Weekday	26	0.035	−0.338 (−0.537 to −0.139)	−0.362 (−0.545 to −0.179)	<0.001	504
**Q-BAPHYP total**	Social media	Weekend	28	0.034	−0.254 (−0.460 to −0.047)	−0.180 (−0.369 to 0.010)	0.064	502
**PedsQL total**	Study/school	Weekday	33	0.049	−0.206 (−0.556 to 0.143)	−0.122 (−0.406 to 0.162)	0.398	497
**PedsQL total**	Study/school	Weekend	33	0.048	−0.148 (−0.465 to 0.169)	−0.170 (−0.439 to 0.099)	0.215	497
**PedsQL total**	Job/internship	Weekday	30	0.047	−0.574 (−0.967 to −0.181)	−0.414 (−0.730 to −0.098)	0.01	500
**PedsQL total**	Job/internship	Weekend	33	0.057	−0.378 (−0.775 to 0.019)	−0.286 (−0.574 to 0.001)	0.051	497
**PedsQL total**	Entertainment	Weekday	30	0.078	−0.562 (−1.007 to −0.116)	−0.485 (−0.811 to −0.159)	0.004	500
**PedsQL total**	Entertainment	Weekend	30	0.129	−0.417 (−0.801 to −0.033)	−0.402 (−0.665 to −0.138)	0.003	500
**PedsQL total**	Gaming	Weekday	31	0.117	−0.285 (−0.759 to 0.189)	−0.309 (−0.610 to −0.008)	0.044	499
**PedsQL total**	Gaming	Weekend	32	0.089	−0.376 (−0.751 to −0.002)	−0.402 (−0.659 to −0.146)	0.002	498
**PedsQL total**	Social media	Weekday	31	0.079	−0.471 (−0.815 to −0.127)	−0.430 (−0.664 to −0.196)	<0.001	499
**PedsQL total**	Social media	Weekend	31	0.086	−0.506 (−0.839 to −0.174)	−0.475 (−0.706 to −0.244)	<0.001	499

Main-model N = 530. Observations with Cook's D >4/530 were excluded only for sensitivity refitting and remained in the main analyses. Each model includes one screen-use exposure and adjusts for age, sex, residence, educational level, and continuous BMI. B denotes outcome-score difference per additional hour/day. Confidence intervals and sensitivity p values are pointwise and not FDR-adjusted.
