# Supplementary material for: Screen Culture’s Association with Generation Z Adolescents’ Posture and Well-Being in Egypt: Cross-Sectional Study
Source: Healthcare (Basel). 2026 Oct 18;14(20):3498. doi: 10.3390/healthcare14203498 (PMC13704529; doi:10.3390/healthcare14203498)
Supplement: Supplementary file 1 [file healthcare-14-03498-s001.zip › Supplementary Table S7.pdf]

Supplementary Table S7. Sensitivity of Activity-Specific Regression Estimates to Self-Reported 24-Hour Screen-Use Values

Outcome	Activity	Period	Reports of 24 h, n	Main B (95% CI)	B after replacing 24 with 20 h/day (95% CI)	Sensitivity p	Change in B
**ISI total**	Study/school	Weekend	3	0.118 (0.012 to 0.224)	0.121 (0.012 to 0.229)	0.03	0.003
**ISI total**	Job/internship	Weekday	1	0.218 (0.109 to 0.327)	0.222 (0.111 to 0.333)	<0.001	0.005
**ISI total**	Job/internship	Weekend	1	0.113 (0.003 to 0.223)	0.114 (0.002 to 0.227)	0.046	0.001
**ISI total**	Entertainment	Weekday	1	0.232 (0.097 to 0.367)	0.241 (0.109 to 0.372)	<0.001	0.009
**ISI total**	Entertainment	Weekend	1	0.161 (0.042 to 0.279)	0.164 (0.044 to 0.284)	0.008	0.003
**ISI total**	Gaming	Weekday	1	0.058 (−0.080 to 0.197)	0.064 (−0.073 to 0.201)	0.36	0.006
**ISI total**	Gaming	Weekend	3	0.079 (−0.033 to 0.190)	0.074 (−0.038 to 0.186)	0.197	−0.005
**ISI total**	Social media	Weekday	6	0.176 (0.077 to 0.275)	0.188 (0.086 to 0.291)	<0.001	0.012
**ISI total**	Social media	Weekend	7	0.209 (0.106 to 0.311)	0.220 (0.113 to 0.327)	<0.001	0.011
**Q-BAPHYP total**	Study/school	Weekend	3	−0.082 (−0.378 to 0.214)	−0.066 (−0.366 to 0.233)	0.664	0.016
**Q-BAPHYP total**	Job/internship	Weekday	1	−0.106 (−0.388 to 0.175)	−0.115 (−0.400 to 0.170)	0.427	−0.009
**Q-BAPHYP total**	Job/internship	Weekend	1	0.067 (−0.192 to 0.327)	0.064 (−0.201 to 0.328)	0.637	−0.004
**Q-BAPHYP total**	Entertainment	Weekday	1	−0.414 (−0.731 to −0.098)	−0.426 (−0.748 to −0.103)	0.01	−0.011
**Q-BAPHYP total**	Entertainment	Weekend	1	−0.413 (−0.730 to −0.095)	−0.430 (−0.742 to −0.118)	0.007	−0.017
**Q-BAPHYP total**	Gaming	Weekday	1	−0.304 (−0.585 to −0.023)	−0.316 (−0.596 to −0.037)	0.027	−0.012
**Q-BAPHYP total**	Gaming	Weekend	3	−0.168 (−0.427 to 0.091)	−0.174 (−0.446 to 0.098)	0.209	−0.006
**Q-BAPHYP total**	Social media	Weekday	6	−0.338 (−0.537 to −0.139)	−0.363 (−0.574 to −0.153)	0.001	−0.026
**Q-BAPHYP total**	Social media	Weekend	7	−0.254 (−0.460 to −0.047)	−0.266 (−0.487 to −0.045)	0.018	−0.012
**PedsQL total**	Study/school	Weekend	3	−0.148 (−0.465 to 0.169)	−0.173 (−0.496 to 0.150)	0.294	−0.025
**PedsQL total**	Job/internship	Weekday	1	−0.574 (−0.967 to −0.181)	−0.560 (−0.946 to −0.175)	0.004	0.013
**PedsQL total**	Job/internship	Weekend	1	−0.378 (−0.775 to 0.019)	−0.361 (−0.748 to 0.026)	0.067	0.017
**PedsQL total**	Entertainment	Weekday	1	−0.562 (−1.007 to −0.116)	−0.582 (−1.028 to −0.137)	0.011	−0.021
**PedsQL total**	Entertainment	Weekend	1	−0.417 (−0.801 to −0.033)	−0.435 (−0.816 to −0.055)	0.025	−0.018
**PedsQL total**	Gaming	Weekday	1	−0.285 (−0.759 to 0.189)	−0.313 (−0.768 to 0.143)	0.178	−0.028
**PedsQL total**	Gaming	Weekend	3	−0.376 (−0.751 to −0.002)	−0.365 (−0.738 to 0.008)	0.055	0.012
**PedsQL total**	Social media	Weekday	6	−0.471 (−0.815 to −0.127)	−0.472 (−0.818 to −0.127)	0.007	−0.002
**PedsQL total**	Social media	Weekend	7	−0.506 (−0.839 to −0.174)	−0.492 (−0.835 to −0.148)	0.005	0.015

Each model includes 530 participants and adjusts for age, sex, residence, educational level, and continuous BMI. Only exposures containing reported 24-hour values were refitted; weekday study/school-related use contained no such reports. Replacing 24 with 20 h/day is a sensitivity scenario, not a validated correction of participants' responses. Counts overlap across activities and should not be summed to obtain the number of affected participants. Change in B is calculated from unrounded estimates and may differ slightly from subtraction of the displayed rounded values. Confidence intervals and sensitivity p values are pointwise and not FDR-adjusted.
